# A homopolymeric adenosine tract in the promoter region of *nspA* influences factor H-mediated serum resistance in *Neisseria meningitidis*

**DOI:** 10.1038/s41598-019-39231-0

**Published:** 2019-02-25

**Authors:** Heike Claus, Kerstin Hubert, Dörte Becher, Andreas Otto, Marie-Christin Pawlik, Ines Lappann, Lea Strobel, Ulrich Vogel, Kay Johswich

**Affiliations:** 1 Institute for Hygiene and Microbiology, University of Würzburg, Würzburg, Germany; 2 Institute for Microbiology, University of Greifswald, Greifswald, Germany

## Abstract

Although usually asymptomatically colonizing the human nasopharynx, the Gram-negative bacterium *Neisseria meningitidis* (meningococcus) can spread to the blood stream and cause invasive disease. For survival in blood, *N. meningitidis* evades the complement system by expression of a polysaccharide capsule and surface proteins sequestering the complement regulator factor H (fH). Meningococcal strains belonging to the sequence type (ST-) 41/44 clonal complex (cc41/44) cause a major proportion of serogroup B meningococcal disease worldwide, but they are also common in asymptomatic carriers. Proteome analysis comparing cc41/44 isolates from invasive disease versus carriage revealed differential expression levels of the outer membrane protein NspA, which binds fH. Deletion of *nspA* reduced serum resistance and NspA expression correlated with fH sequestration. Expression levels of NspA depended on the length of a homopolymeric tract in the *nspA* promoter: A 5-adenosine tract dictated low NspA expression, whereas a 6-adenosine motif guided high NspA expression. Screening German cc41/44 strain collections revealed the 6-adenosine motif in 39% of disease isolates, but only in 3.4% of carriage isolates. Thus, high NspA expression is associated with disease, but not strictly required. The 6-adenosine *nspA* promoter is most common to the cc41/44, but is also found in other hypervirulent clonal complexes.

## Introduction

*Neisseria meningitidis* causes invasive meningococcal diseases (IMD) such as meningitis or septicaemia predominantly in infants and toddlers, but also in adolescents. Pathogenic meningococci express a polysaccharide capsule, which protects them against killing by the complement system^1^. The chemical composition of the capsule defines twelve distinct serogroups^2^, of which serogroup B dominates the *N. meningitidis* epidemiology in the Northern Hemisphere^3^. Serogroup B meningococci causing invasive disease usually belong to few so-called ‘hypervirulent’ clonal complexes (cc). The sequence type (ST-) 41/44 clonal complex (cc41/44), a major cc among disease causing serogroup B meningococci, caused several epidemics in the 1980s and 1990s in the Netherlands and in New Zealand, respectively^3^. The cc41/44 is characterized by a surprisingly homogeneous repertoire of surface antigens, but on the contrary, it comprises a huge number of individual sequence types, which are defined based on the sequences of seven different housekeeping genes (multilocus sequence typing, MLST)^4^. Some of the STs within cc41/44 are exclusively associated with asymptomatic carriage, whereas others can also cause invasive disease^5,6^. The underlying genetic or antigenic mechanisms determining whether a given ST is associated with either carriage or disease are widely unknown.

Resistance to the lytic activity of human serum is necessary for invasiveness of *N. meningitidis* isolates^1^ and is almost entirely attributable to the polysaccharide capsule. Yet, in addition to the capsule, several other surface components have been demonstrated to enhance serum resistance. These factors comprise sialylation of the meningococcal lipooligosaccharide (LOS)^7,8^, the factor H binding protein (fHbp, formerly called GNA1870)^9,10^, the *Neisseria* heparin binding antigen (NHBA, formerly called GNA2132)^11^ and the *Neisserial* surface protein A (NspA, NMB0663)^12,13^.

The outer membrane proteins fHbp and NHBA are components of the multi-component serogroup B vaccine (Bexsero)^14^. fHbp directly sequesters the complement regulator of the alternative pathway, factor H (fH), thereby reducing deposition of C3b to the meningococcal surface and intercepting the amplification feedback loop of complement activation^9,15^. NHBA binds to heparin, thereby increasing cellular attachment^16^; additionally, NHBA has also been reported to enhance serum resistance of unencapsulated *N. meningitidis*^11^. This has been suggested to occur via indirect fH recruitment, which interacts with heparin^17^. However, in presence of capsule, no NHBA-dependent increase of serum resistance was seen^11,18^.

NspA can directly recruit fH to the bacterial surface to limit C3b deposition and thereby intercepts the positive feedback loop of the alternative pathway, leading to increased serum resistance of *N. meningitidis*^12,13^. NspA is a highly conserved surface protein in *N. meningitidis* and *N. gonorrhoeae*^19,20^, but is expressed in only few commensal *Neisseria* species^20^. The structure of the outer membrane protein has been resolved as an eight-stranded antiparallel β-barrel with four extracellular loops^21^. NspA expression is iron activated, and the activation is triggered by Fur dependent and independent mechanisms^22^. Furthermore, mutation of the anti-sigma factor MseR reduces expression of nspA^23^, and levels of NspA in outer membrane vesicles depend on growth media conditions^24^. NspA has been proposed as vaccine candidate, since mice immunized with recombinant NspA show protection against experimental infection^20^ and protective antibodies targeting NspA were elicited during experimental infections of mice^25^. A variety of bactericidal monoclonal antibodies directed against NspA has been published^26–28^. Nevertheless, presentation of the antigen in outer membrane vesicle-derived vaccine (strains H44/76 and NZ98/254) did not elicit measurable anti-NspA antibodies in humans^29^. Similarly, a recombinant meningococcal NspA vaccine did not induce a bactericidal response in humans^30^. In the experimental mouse system, NspA was not critical for virulence of *N. meningitidis*^27^, a finding to be considered with care, because fH-recruitment by NspA is species-specific^13^. Furthermore, there is compelling evidence that NspA protects bacteria from serum killing by recruitment of the complement regulator fH^12,13^.

In this study we conducted proteome analyses on closely related *N. meningitidis* strains of cc41/44 isolated from IMD cases versus asymptomatic carriage which displayed distinct degrees of serum resistance. NspA was more abundant in the invasive isolate compared to both carriage isolates. Deletion of *nspA* in the invasive strain increased complement deposition to the level of the carrier strain. The length of a poly(A) tract of the *nspA* promoter correlated with expression levels of NspA, and *N. meningitidis* with high NspA expression displayed reduction of membrane attack complex insertion and increased serum resistance.

## Results

### Difference in serum resistance of disease versus carriage isolates of cc41/44

**Figure 1:**
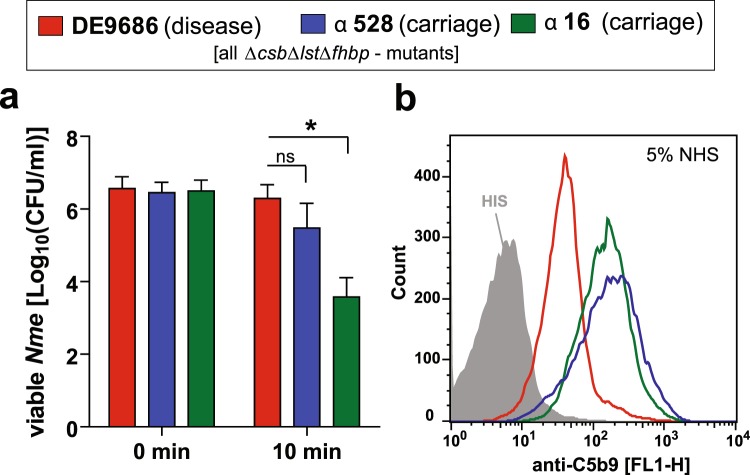
Differential serum resistance in closely related *N. meningitidis* cc41/44 strains lacking capsule, fHbp and LOS sialylation. (**a**) Survival of *N. meningitidis* for 10 min in 5% NHS assessed by dilution plating. In all strains, established factors of complement evasion such as capsule synthesis (Δ*csb*), factor H binding (Δ*fhbp*) and LOS sialylation (Δ*lst*) were deleted. The corresponding parental strains had been isolated from invasive disease cases (DE9686) or from asymptomatic carriers (α528, α16). The graph plots mean ± SEM from three independent experiments. * denotes *P* < 0.05 in one way ANOVA applying Dunnett’s *post hoc* test using DE9686 as reference. (**b**) Deposition of C5b9 on *N. meningitidis* isolates as in (**a**) as assessed by flow cytometry. One out of three independent experiments with similar results is shown.

Meningococci of the cc41/44 are responsible for a large proportion of serogroup B disease worldwide but are also frequent among healthy carriers^5,6^. We selected three genetically related meningococcal strains, one disease isolate (DE9686) and two carrier isolates (α16 and α528), to investigate their resistance towards human serum. For our analysis, triple mutants were generated which lacked capsule, LOS sialylation and fHbp, thereby excluding factors of established serum resistance mechanisms, in order to focus on novel or less well perceived factors. The mutant of the disease strain (DE9686Δ*csb*Δ*lst*Δ*fHbp*) was more resistant to a challenge with 5% normal human serum (NHS) than those of the two carrier isolates (α16Δ*csb*Δ*lst*Δ*fHbp* and α528Δ*csb*Δ*lst*Δ*fHbp*) and showed less C5b9 deposition in presence of 5% NHS (Fig. 1). This finding indicates that factors other than capsule, LPS and fHbp might contribute to serum resistance of the disease isolate.

### Higher NspA expression in the disease isolate belonging to cc41/44

**Figure 2:**
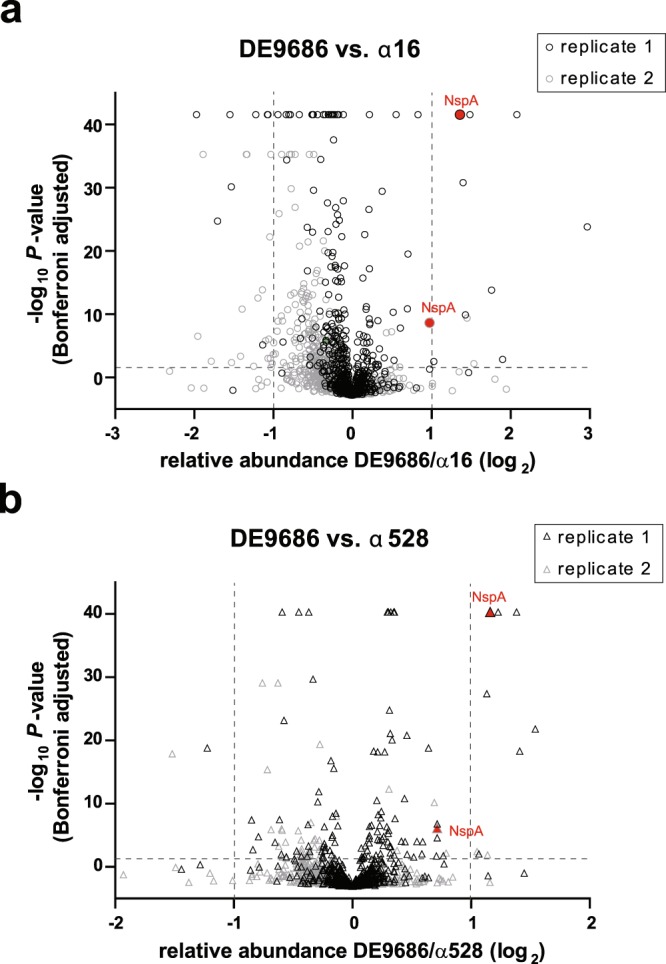
Proteome analysis by LC-MS/MS. Volcano plots depict the relative abundance of individual proteins (plotted as log_2_ ratio on x-axis) comparing strains DE9686 with α16 (**a**) or DE9686 with α528 (**b**). Y-axis plots the Bonferroni-adjusted *P-*value for each individual protein differentially expressed between the disease and the corresponding carrier strain. For each strain comparison, two independent experiments (replicates) were performed, indicated by grey and black symbols. Due to variances between the two independent replicates, NspA was identified as the only protein to be consistently and significantly higher expressed in strain DE9686 in both comparisons (highlighted by red symbols).

In order to identify the factor responsible for higher serum resistance, comparative whole cell proteome analysis of the three strains was conducted using a LC-MS/MS approach, which identified a higher expression of NspA in the invasive isolate compared to both carrier isolates. The volcano plots shown in Fig. 2 combine the data derived from two biologically independent replicates for each of the two strain pairs (DE9686 vs. α16 and DE9686 vs. α528, respectively). Despite some variation among the biological replicates, NspA protein was consistently more abundant in strain DE9686 in all four settings. A list of all proteins with different abundance in both replicates for each strain comparison is shown in Supplementary Table S1. As the only consistent hit from the proteome analysis, NspA was analyzed in detail in further experiments.

**Figure 3:**
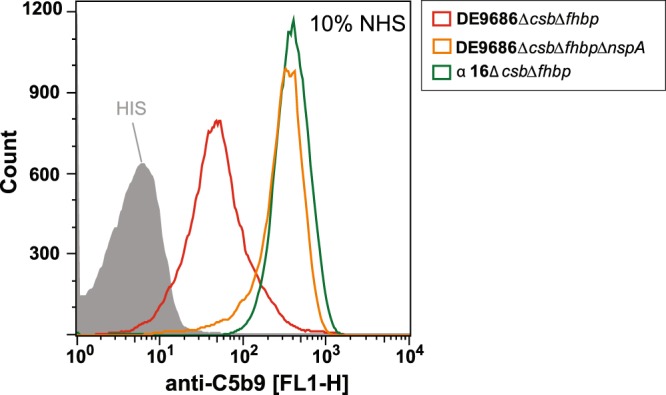
Deletion of *nspA* enhances C5b9 deposition onto *N. meningitidis*. Meningococcal strains were incubated in 10% NHS for 10 min and then C5b9 deposition was assessed by flow cytometry. Notably, when *nspA* was deleted from DE9686, it showed similar C5b9 deposition as the α16 strain. All strains used in this experiment were devoid of capsule and fHbp. The graph plots one of three experiments with similar outcome.

Indeed, when the *nspA* gene in the disease strain DE9686 was deleted, this led to an increase of complement C5b9 surface deposition to a level comparable to that of the carriage strain α16, when incubated with 10% NHS (Fig. 3). These data suggest that NspA participates in the protection against complement in cc41/44 disease isolates.

### A single basepair difference in a homopolymeric poly(A) tract in the promoter region of *nspA* gene determines NspA expression levels in disease isolates

**Figure 4:**
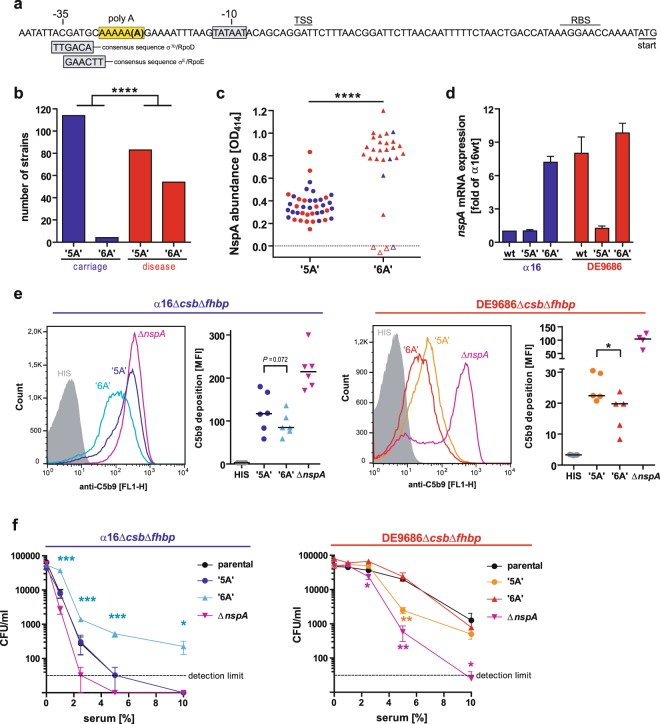
A homopolymeric tract in the *nspA* promoter guides NspA protein expression. (**a**) Schematic representation of the *nspA* promoter region with indicated −10 box (Pribnow, TATA), deduced transcriptional start site (TSS), ribosomal binding site (RBS) and start codon. A poly(A) tract containing either 5 or 6 adeonsine (A) residues upstream of the -10 box is highlighted in yellow. The position and consensus sequence of −35 elements known for other *N. meningitidis* promoters recognized by σ^70^ (RpoD) or σ^E^ (RpoE) are indicated below. (**b**) Proportion of homopolymeric tracts featuring ‘5A’ or ‘6A’ in the *nspA* promoter of 118 carriage strains (blue) versus 137 disease strains (red) of cc41/44. **** Indicates *P* < 0.0001 by Fisher’s exact test. Details on the analyzed strains are listed in Supplementary Table S2. (**c**) NspA protein expression in a collection of disease strains (red) and carrier strains (blue) as assessed by whole cell ELISA in dependence of homopolymeric tract length (‘5A’ (circles) versus ‘6A’ (triangles)). Open symbols indicate strains with a frameshift mutation within the *nspA* gene. **** Indicates *P* < 0.0001 in unpaired, two-tailed Student’s T-test. (**d**) qRT-PCR analysis of *nspA* transcription of α16 and DE9686 either in the wild-type strain or in mutants in which the homopolymeric tract was mutated to yield either a ‘5A’ or ‘6A’ sequence. Note that ‘wt’ here refers to the *nspA* gene; the strains are mutants lacking *csb* and *fhbp*. (**e**) C5b9 deposition onto *N. meninigitidis* strains (left panels: α16; right panels: DE9686; both strains are deletion mutants lacking capsule and fHbp) featuring either a ‘5A’ or a ‘6A’ homopolymeric tract in the promoter region or deletion of *nspA*. Histograms are representative of a total of 5 or 6 independent experiments; graphs plot the mean fluorescence intensity (MFI) of the corresponding experiments. * Indicates *P* < 0.05 in unpaired, two-tailed Student’s T-test. (**f**) Survival in normal human serum of *N. meninigitidis* strains (left panels: α16; right panels: DE9686; both strains are deletion mutants lacking capsule and fHbp) featuring either a ‘5A’ or a ‘6A’ homopolymeric tract in the promoter region or deletion of *nspA*. Plotted are means ± SEM of n = 4–6 independent experiments. *, **, *** Denote *P* < 0.05 or 0.01 or 0.001 in one-way ANOVA applying Dunnett’s *post hoc* test (comparison against the parental strain).

In searching for the underlying reason for the differential expression of NspA in carriage versus disease strains of cc41/44, we compared the coding and promoter regions of the *nspA* genes of our test strains. While the NspA amino acid sequence was identical among the three strains, the promoter analysis revealed a sequence difference in a homopolymeric poly(A) tract upstream of the Pribnow-box (−10 box or TATA box) of the promoter, featuring 5 consecutive adenosines (‘5A’) in the carriage isolates but 6 adenosines (‘6A’) in the disease isolate (Fig. 4a). This finding prompted us to analyze this sequence motif in a collection of German cc41/44 strains from invasive disease cases (n = 137) collected at the National Reference Laboratory for Meningococci and Haemophilus influenzae (NRZMHi) in Würzburg from 2001 to 2010 (further referred to as ‘disease strain collection’) and carriage strains (n = 118) isolated in Bavaria in the winter season 1999/2000^31^ (further referred to as ‘carriage strain collection’). The respective strains of both collections and their characteristics are listed in Supplementary Table S2. There was a striking difference in the frequency of poly-A tracts with 6 adenosines in the *nspA* promoter region among disease versus carriage strains: While 39% of disease isolates featured a ‘6A’-homopolymeric tract, this was only seen in 3.4% of carriage isolates (Fig. 4b). In addition, there were some sequence differences among the strains at positions −6 (A/C), +1 (G/A) and +12 (G/A), which did not correlate with *nspA* expression and were not further analyzed. By whole cell ELISA, we found that NspA protein is stronger expressed in strains with a ‘6A’-tract; *vice versa* NspA is less abundant in strains with a ‘5A’- tract (Fig. 4c). Our analysis identified four strains with a ‘6A’-tract that had no detectable levels of NspA, however, these strains featured a deletion at nucleic acid position 193 leading to a frameshift yielding a premature stop codon. In order to confidently attribute NspA expression levels to the number of adenosine residues in the homopolymeric tract upstream of the −10 box, we constructed isogenic mutants of DE9686 and α16 which carried either ‘5A’- or ‘6A’-tracts in the *nspA* promoter region. Indeed, relative mRNA expression of *nspA* in both genetic backgrounds was strictly guided by the number of adenosines in the homopolymeric tract, with ‘6A’ leading to high and ‘5A’ to low *nspA* mRNA quantities as analyzed by qRT-PCR (Fig. 4d). In accordance with low NspA expression in the ‘5A’ mutants of either α16 or DE9686, the bacteria showed high levels of C5b9 deposition after serum exposure; *vice versa*, when a ‘6A’-tract was introduced, levels of C5b9 deposition declined (Fig. 4e). Furthermore, the ‘6A’-tract bearing mutants (lacking capsule and *fhbp*) showed significantly higher survival in normal human serum, whereas deletion of *nspA* reduced their serum resistance (Fig. 4f).

### Significance of ‘5A’ or ‘6A’ NspA versus fHbp in fH-recruitment by cc41/44 strains

**Figure 5:**
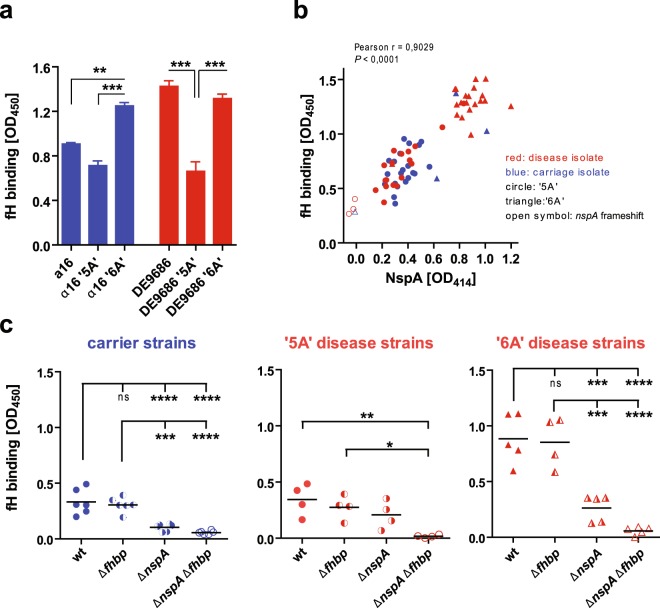
fH binding to *N. meningitidis* strains correlates with NspA protein abundance. (**a**) Whole-cell ELISA-based fH binding to wild-type strains of carriage isolate α16 and disease isolate DE9686 and their corresponding mutants featuring either a ‘5A’ or ‘6A’ motif in the *nspA* promoter. *, **, *** Denote *P* < 0.05 or 0.01 or 0.001 in one-way ANOVA applying Bonferroni’s *post hoc* test. (**b**) Correlation of NspA protein expression and fH binding as assessed by whole cell-ELISAs. For statistical analysis, Pearson’s correlation was used. (**c**) fH-binding to cc41/44 isolates from carriers (α16, α80, α253, α528, α547, α726), disease strains featuring a ‘5A’ *nspA* promoter (DE8794, DE9492, DE10829, DE11216) or a ‘6A’ *nspA* promoter (DE8658, DE9686, DE9129, DE10470, DE11204) and their corresponding mutants lacking *fHbp*, *nspA*, or both. Statistical testing was done using one-way ANOVA applying Bonferroni’s *post hoc* test; *, **, ***, **** Denote *P* < 0.05 or 0.01 or 0.001 or 0.0001, respectively. ns, not significant.

Since *nspA* genes with a ‘6A’ homopolymeric tract lead to enhanced expression of NspA protein and reduced C5b9 deposition onto the cells, we wanted to test whether this effect is due to increased sequestration of fH to the bacterial surface. In fact, when the α16 strain and the DE9686 strain featuring either the ‘5A’ or the ‘6A’ motif were tested for fH binding, there was a clear down- or up-shift, respectively, in fH surface sequestration (Fig. 5a). Moreover, we probed fH-binding to the entire strain collection, for which NspA protein levels were known (Fig. 4b), by a whole-cell fH binding assay. Indeed, there was a striking correlation between NspA abundance and fH binding (Fig. 5b). Yet, there still was notable fH binding to *N. meninigitidis* strains which lacked NspA protein due to a frameshift mutation in the *nspA* coding region (indicated by the open symbols).

This NspA-independent fH binding is most likely due to fHbp, but we were surprised that this effect was so low in comparison to the NspA-dependent fH binding. In order to compare the relative binding of fH to either NspA or fHbp, we generated mutants of carriage strains, ‘5A’ disease strains and ‘6A’ disease strains which lacked either or both of these fH-binding proteins and subjected them to the fH binding assay. In fact, deletion of *nspA* had a more pronounced effect than deletion of *fHbp*, which only marginally reduced fH binding to the cc41/44 strains analyzed here (Fig. 5c). Throughout, deletion of both genes almost entirely ablated fH binding. Presence or absence of fHbp and NspA protein in the mutants coated on ELISA plates was ascertained with either mAb clone 14C7 (anti-NspA)^32^ or JAR 41 (anti-fHbp, recognizing all fHbp variant groups)^33^. We ruled out a significant differential effect of capsulation on fH-binding to the strains used here by assessing capsule expression using whole-cell ELISA and plotted it against the fH-binding (Supplementary Fig. S1).

From these data we conclude that in cc41/44 strains, fH binding is chiefly attributable to NspA, whereas fHbp contributes to a minor proportion of the fH surface sequestration. Thus, variances of NspA expression precipitate stark differences in the ability of these strains to evade complement via fH binding.

### No evidence for phase variation of nspA by variable length of poly(A) stretch in promoter

Phase variation is a mechanism in *N. meningitidis* to stochastically diversify a growing culture with respect to immunogenic structures of the outer membrane, thereby allowing evasion of adaptive immune responses by the host. Aside from the mechanisms of gene conversion altering the coding sequence of a protein (*e.g. pilE*/*pilS*)^34^ and slipped strand mispairing within the coding sequence of a gene switching functional protein translation ‘on’ or ‘off’ (*e.g. opa*)^35^, variations in the length of homopolymeric tracts in the promoter region can tune gene transcription, as has been demonstrated for the *opc* gene^36,37^. Based on our findings, we suspected that expression of the *nspA* gene might be subject to phase variation, guided by the length of the poly(A) sequence upstream of the −10 box.

In order to test for phase variation of *nspA*, strain DE9686ΔfHbpΔcsbΔmutS-‘5A’ was generated, which is susceptible to serum killing (lacking capsule and fHbp) and contains a ‘5A’ homopolymeric tract in the promoter. The mismatch repair gene *mutS* was deleted in order to enhance the likelihood of spontaneous mutations increasing phase variation^36,38^. This test strain was repeatedly exposed to 5% NHS, a condition leading to a consistent reduction of viable counts by 4 orders of magnitude. Of 66 surviving clones in the third round of serum challenge, the poly(A) tract in the *nspA* promoter was analyzed by sequencing, yielding not a single event of phase variation back to the ‘wild-type’ sequence of the parental DE9686 strain featuring ‘6A’. As a control, the poly(C) tract in the *opc* promoter from recovered clones of the same experiments was analyzed in parallel, displaying a shift from ‘11C’ to ‘12C’ or ‘14C’ in 5 of 17 analyzed clones, consistent with our earlier study^36^. Thus, we could not detect phase variation of *nspA* in our setting. While this does not entirely exclude the possibility of phase variation, it suggests that if phase variation of this gene occurs at all, it only does so at a very low frequency and with a *mutS* independent mechanism.

### Differential distribution of poly(A) tract length among sequence types within cc41/44

**Figure 6:**
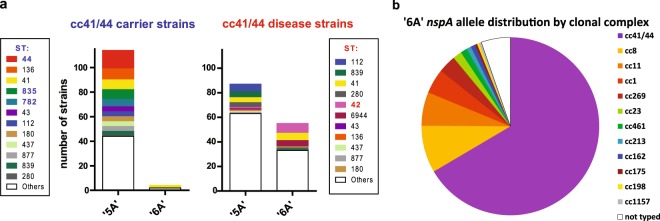
Epidemiology of ‘5A’ and ‘6A’ bearing carriage and disease isolates. (**a**) The *nspA* promoter region of cc41/44 strains (n = 255; see Supplementary Table S2) from the ‘carriage strain collection’ and the ‘disease strain collection’ was analyzed for the number of adenosines in the homopolymeric tract upstream of the −10 box, and the according ST’s of the individual strains plotted in the diagram. Plotted with individual colours are all ST’s for which at least 3 strains were found in one out of the four conditions presented in the entire figure (carriage versus disease and ‘5A’ versus ‘6A’); all other hits were combined as ‘others’. Blue highlighted ST’s indicate those only found among carriage isolates (n = 118), red highlighted ST’s were only found in disease isolates (n = 137). (**b**) Occurence of the ‘6A’ motif in various clonal complexes of *N. meningitidis* strains as found by BLAST search of all available meningococcal genome sequences in the PubMLST database (https://pubmlst.org/neisseria/). Data are expressed as percentage of all ‘6A’ bearing isolates (n = 244) found by the database search (see Supplementary Table S3).

We analyzed whether the poly(A) tract length of strains in our ‘disease strain collection’ and our ‘carriage strain collection’ (as mentioned above) is linked to certain sequence types within cc41/44. In general, there was an evenly scattered distribution of the individual ST’s among carrier strains and disease strains, albeit a few exceptions were found (Fig. 6a). In the strain collections analyzed here, ST-44, ST-835 and ST-782 were only represented in carriers (which almost exclusively feature a ‘5A’ homopolymeric tract) but not in invasive disease, whereas ST-42 was only represented in invasive disease but not in carriers. Notably, among invasive disease isolates, ST-112 was the most abundant ‘5A’ bearing ST, but was not found among the ‘6A’ bearing isolates. *Vice versa*, ST-42 isolates had mostly a ‘6A’ genotype and only few isolates from ST-42 harbored the ‘5A’ motif. Similarly, ST-6944 was exclusively found with a ‘6A’ phenotype. In contrast to the aforementioned, ST-41 was found in carriage as well as disease strains, either harboring a ‘5A’ or a ‘6A’ motif. Although our analysis considered quite a large number of ST-41/44 isolates, the data must be interpreted with care, since there was a large number of unique ST’s, diluting the actual number of strains detected from each ST. In addition, it is pertinent to note that the carriage isolates and the disease isolates were collected at different times and geographically not entirely overlapping, as mentioned above. Thus, larger studies are required to confirm our findings.

### ‘6A’ motif not restricted to, but most common in cc41/44

We wondered whether the ‘6A’ motif in the *nspA* promoter is a feature restricted to the cc41/44 or whether it is commonly found in other clonal complexes as well. Therefore, we did a BLAST search (performed July 2018) of all publicly available *Neisseria meningitidis* sequences in the PubMLST database (https://pubmlst.org/) and screened for the ‘6A’ *nspA* promoter motif. We found a total of 244 genome-sequenced meningococcal isolates harboring this motif (Supplementary Table S3). Of these strains, 66.5% belonged to cc41/44, 8.6% to cc8, 6.1% to cc11, 4.5% to cc1 and 3.3% to cc269 (Fig. 6b). Notably, we did not find a single isolate with a ‘6A’ *nspA* promoter from the epidemiologically relevant hypervirulent cc32, which accounts for ~20% of invasive disease cases in Europe according to the 2012 ECDC Surveillance report of invasive bacterial diseases^39^. Thus, the ‘6A’ *nspA* promoter motif is most common in cc41/44 strains, but it is also found in other significant hypervirulent clonal complexes of *N. meningitidis*. However, not all hypervirulent clonal complexes (*e.g*. cc32) seem to harbor the ‘6A’ *nspA* promoter motif.

## Discussion

The capacity of a meningococcal strain to cause invasive meningococcal disease is a polygenic trait, and it is hitherto unclear which (or whether at all) discrete genetic determinants define whether meningococcal strains remain as harmless colonizers in the nasopharyngeal mucosa or whether they become invasive. The most important feature determining their virulence is the ability to resist complement killing, allowing their survival and multiplication in the blood^1^. This is chiefly attributable to their polysaccharide capsule. Hence, the lack of capsule clearly determines the non-pathogenicity of a given meningococcal strain; in fact, many meningococci found in carriers are unencapsulated and therefore non-pathogenic^31^, with very few exceptions^40^. On the contrary, the vast majority of encapsulated meningococcal strains do precipitate in disease, thus, the capsule is a necessary, but not sufficient prerequisite for invasive disease.

Aside from the capsule, the outer membrane protein fHbp aids in complement evasion by sequestration of the complement regulatory protein fH, which functionally limits complement deposition onto the meningogoccal surface^9,10,41^. In addition, NspA has been described as receptor for fH, which as well limits complement deposition^12,13^. Noteworthy, the contribution of fHbp and NspA to meningococcal serum resistance is significantly less as compared to that of the capsule. While fHbp has been under intense investigation, leading to its use as vaccine component^14,42^, NspA has attracted less attention thus far.

In our study, we focused on serogroup B strains of cc41/44 as a model for genetically similar *N. meningitidis* strains which can either be limited to carriage or precipitate invasive disease. Our analyses revealed a striking difference in the NspA surface expression levels between carriage and invasive disease isolates. However, it is pertinent to note that our analyses regarding C5b9-deposition or survival in serum (Figs 1, 3 and 4) were done using mutants lacking capsule and fHbp. This was necessary to identify serum resistance factors aside from the dominant and well described capsule or fHbp. However, many carriage strains are naturally unencapsulated, thereby possibly relying mostly on their surface proteins to enable a potentially required level of complement evasion within the mucosal niche.

In general, surface-exposed proteins of *N. meningitidis* are subject to a high evolutionary pressure to escape the adaptive immune system, which often results in high antigenic variation of homologous proteins among different strains^43^. Yet, NspA shows a remarkably high level of amino acid sequence conservation^44^. In this case, modulation of protein abundance on the bacterial surface can fine-tune the balance between the benefit of protein function and the disadvantage of antibody recognition. Variation of protein expression is often induced by phase variation, a process in which oligonucleotide repeats or homopolymeric tracts either within the promoter or in the coding sequence of a given gene are stochastically varied upon replication^45^. When the promoter is affected, this results in changes in the transcription efficiency and up- or down-regulation of protein. When the coding region is affected, this often leads to a premature stop-codon, leading to an ON or OFF state of the protein expression. Our analyses revealed that there is a subtle difference in a homopolymeric adenosine tract of the *nspA* promoter in cc41/44 strains. Carriage strains almost exclusively harbored a ‘5A’ motiv, driving a relatively low expression of NspA on the bacterial surface. In contrast, 39% of invasive disease strains harbored a ‘6A’ tract, governing high expression. In fact, it was noted in previous studies that NspA expression levels vary significantly among genetically diverse meningococcal strains of serogroup B^44^, and our results now provide a mechanistic explanation for this fact, at least within the cc41/44. However, our experiments to detect phase variation did not yield a single event where a switch from ‘5A’ to ‘6A’ occured. Thus, the two sequence motifs ‘5A’ and ‘6A’ are either not the result of phase variation, or phase variation occurs at a much lower level *in vitro* as observed for other ‘classically’ phase variable genes such as *opc*. In fact, phase variation and mutations in short repetitive nucleotide tracts in bacterial genomes generally occur at a much lower rate than in longer homopolymeric tracts. The ‘6A’ motif is much more common in disease isolates than in carriage isolates, however, the fact that ~60% of the disease strains still feature a ‘5A’ tract implies that high expression of NspA on its own is not a prerequisite for disease, while its low expression does not determine non-invasiveness. Still, we conclude that a ‘6A’ *nspA* is strongly associated with invasive disease.

It was our first notion that the ‘5A’/’6A’ motif might differentially space apart the −10 and the −35 sequence common to many promoters, *e.g*. in *E. coli*. However, the *nspA* promoter does not harbor a −35 consensus sequence (TTGACA), which enhances σ^70^ (RpoD in *Neisseria*) dependent gene transcription. In fact, promoters in pathogenic *Neisseria* only seldomly contain a −35 consensus sequence^46,47^. Thus, the spacing of −35 and −10 regions can be ruled out as explanation for the differential *nspA* expression between the ‘5A’ and ‘6A’ genotypes. In absence of a −35 sequence, σ^70^ dependent transcription is enhanced by an extended −10 box (consensus: TGxTATAAT); however the *nspA* promoter possesses only a canonical −10 box (TATAAT). The *nspA* promoter also contains no binding motif for the extracytoplasmic sigma factor, σ^E^ (RpoE) and for *N. meningitidis*, there is no consensus sequence known for the second alternative sigma factor, RpoH^46^. Hence, we speculate that *nspA* transcription might require other transcription factors (e.g. Fur), which in turn may act stronger on the ‘6A’ promoter than on the ‘5A’ promoter.

Our database analysis revealed that the ‘6A’ motif in the *nspA* promoter is most common in cc41/44 strains, but it is also found in strains belonging to other hypervirulent clonal complexes such as cc8, cc11, cc1 and cc269. Interestingly, we did not find a single isolate in the PubMLST database with a ‘6A’ *nspA* promotor belonging to cc32, which is the most frequent cause of serogroup B invasive disease cases in Europe together with cc41/44. Of note, cc32 strains such as H44/76 or MC58 do harbor the *nspA* gene (with a ‘5A’ promoter). One caveat of our database analysis worth mentioning is that the results might be biased by the composition of database entries, which is encyclopaedic rather than representative. However, we are confident that the trend observed in our analysis at least roughly reflects the distribution of ‘6A’ *nspA* promoter-harboring (and thus, highly NspA expressing) strains among different clonal complexes.

The high expression of ‘6A’ bearing *nspA* genes was functionally important to recruit higher levels of fH and reducing complement deposition onto the bacteria. In fact, NspA expression guided by either ‘5A’ or ‘6A’ harboring *nspA* promoter was clearly correlated with fH binding, although NspA is only one out of two major proteins of *N. meningitidis* to recruit fH. Our analysis of numerous mutant strains revealed that, for cc41/44 strains, NspA seems to be dominant over fHbp in recruitment of fH, since across all 15 strains analyzed, the deletion of NspA caused a stronger reduction in fH binding than the deletion of fHbp did. Indeed, differing amplitudes for fH recruitment by either of the factors have been described before, as strain H44/76, a high-expresser of fHbp, relies mostly on fHbp to reduce C3-deposition, which is contrasted by strain A2594, which mostly relies on NspA to recruit fH^12^. Further research is required here to put the different contributions of the individual fh-binding proteins of *N. meningitidis* into biological context using whole blood models or *in vivo* models.

In conclusion, the serum resistance of invasive strains of the cc41/44, and putatively also other hypervirulent lineages, is partially determined by a single nucleic acid in the promoter region of the *nspA* gene, driving expression levels and thereby enhancing fH recruitment and complement evasion.

## Materials and Methods

### Meningococcal wild-type strains

Strain DE9686 (B:P1.7–2,4:F1–5) is a clinical isolate from the cerebrospinal fluid of a four-year-old female from Germany that caused meningitis and sepsis. Strains α16 (B:P1.7–2,4:F1–5) and α528 (B:P1.7–2,15–39:F1–5) are non-invasive carrier isolates from a 19-year-old male and a 14-year-old female both from Germany, which were isolated during the Bavarian meningococcal carriage study^31^. All three strains belong to cc41/44 but are assigned to different ST’s, *i.e*. ST-42 (DE9686) and ST-41 (α16 and α528). Characteristics of these strains and mutant strains used in this study are listed in Supplementary Tables S2 and S4, respectively. Further invasive strains used for the analysis of NspA expression were obtained from the German National Reference Laboratory for Meningococci and Haemophilus influenzae (NRZMHi) located at the Institute for Hygiene and Microbiology of the University of Würzburg (www.nrzmhi.de). German carrier isolates were derived from the Bavarian meningococcal carrier study^31^. Characteristics of these additional strains, as well as data regarding their poly-A tract in the *nspA* promoter and OD in NspA protein expression ELISA are listed in Supplementary Table S2.

### Mutant strains

All *N. meninigitidis* strains and mutants are listed in Supplementary Table S4, primer sequences are listed in Supplementary Table S5. The serogroup B polysialyltransferase gene *csb* (formerly *siaD* or *synD*), the lipopolysaccharide sialyltransferase gene *lst*, and the *fhbp* gene were deleted as described previously^8,48,49^.

To delete *nspA*, DNA fragments upstream and downstream of *nspA* were amplified using primers KH145 and KH146 and primers KH147 and KH148, respectively. The PCR products were cloned as flanking sequences of an erythromycin resistance cassette in pBluescript. The resulting plasmid (pAB13) was used for transformation of *N.meningitidis*. Correct recombination into the meningococcal genome was confirmed by DNA sequencing and Southern blot.

Isogenic *nspA* mutants with either 5 adenines or 6 adenines within the nspA promoter were constructed as follows: The entire *nspA* gene together with its promoter region was amplified with primers HC630 and HC631 comprising a homopolymeric tract of five adenine residues (5As) when strain α16 was used as template or of six adenine residues (6As) when strain DE9686 was used as template. The downstream region of *nspA* was amplified with primers HC623 and HC621. Subsequently, a kanamycin resistance cassette was ligated between the PCR products via NdeI and BglII restriction sites. Both ligation products were amplified with primers HC630 and HC621 and the resulting PCR products harboring either five or six adenine residues in the *nspA* promoter were transformed into the appropriate meningococcal strains. Successful recombination was proved by PCR and sequencing.

Other plasmids for deletion of *fHbp* (plasmid pMP-1)^9^, *lst* (pGH7)^8^ or *csb* (formerly *siaD*; pMF32.35::T5)^50^, have been previously published as indicated.

### Reagents

Pooled human complement serum (normal human serum, NHS) was purchased from Innovative Research inc. (USA) via Dunn Labortechnik (Germany). Monoclonal antibody 14C7 (IgG3) is directed against NspA, antibody JAR41 is directed against fHbp; both were kindly provided by Dan M Granoff, Children's Hospital Oakland Research Institute, California, USA^32,33^. The monoclonal antibody mAb B306 used to detect OpcA was a kind gift of Mark Achtman^51^.

### Serum killing assay

*N. meningitidis* were grown overnight at 37 °C, 5% CO_2_ and water saturated atmosphere on GC chocolate agar (Becton Dickinson). 4 × 10^4^ bacteria were incubated in Veronal buffered saline supplemented with MgCl_2_, CaCl_2_ and bovine serum albumin (VBS/BSA: 5 mM Barbital, 145 mM NaCl, 0.5 mM MgCl_2_, 0.15 mM CaCl_2_, 0.5% BSA, pH7.4) and, depending on the assay (as indicated), 1%, 2.5%, 5% or 10% NHS in a final volume of 400 µl at 37C and 700 rpm for 10 minutes. The reaction was stopped by adding 400 µl cold PBS and serial dilutions were plated on blood agar to estimate colony forming units (CFU) in comparison to the inoculum.

### Flow cytometric analysis

Strains were grown on GC chocolate agar plates overnight at 37 °C, 5% CO_2_. 2 × 10^8^ cells were incubated in VBS/BSA with 5% or 10% NHS or heat-inactivated (56 °C at 30 min) NHS (HIS) as negative control for ten minutes at 37 °C while shaking (200 rpm). The reaction was stopped on ice by addition of 400 µl of cold Hank’s Balanced salt solution containing 1 mM Ca^2+^, 0.15 mM Mg^2+^ and 1% BSA (HBSS^++^/BSA). Bacteria were washed twice with HBSS^++^/BSA. Antibody incubations were done in a final volume of 50 µl in HBSS^++^/BSA at 37 °C for 30 minutes and shaking at 700 rpm. Monoclonal antibody anti-C5b9 (clone aE11; Dako cytomation) was used to detect terminal complex of complement deposited onto the *N. meningitidis* surface. After washing, AlexaFluor488 goat anti-mouse IgG (Jackson ImmunoResearch) was used as secondary antibody. Bacteria were fixed in PBS with 1% formaldehyde for 1 h, then pelleted and resuspended in 400 µl PBS before analysing samples on a FACS Calibur (Becton Dickinson).

### Mass spectrometry

*N. meningitidis* were grown for approximately 12 hours on GC chocolate agar plates. Bacterial suspensions with OD_600_ of 5.0 were prepared in 3 ml ice cold 50 mM TEAB (Triethylammoniumbicarbonate, Sigma-Aldrich). Cell disruption by homogenization with glass beads was carried out in four cycles at 6.5 m/s for 45 seconds with intermittent cooling for 1 minute on ice (FastPrep®, MP Biomedicals). Cell debris was removed by centrifugation at 13000 rpm (17226x g) at 4 °C for two minutes and at 14000 rpm (23447x g) for 30 minutes at 4 °C. The supernatant was sterile filtered through a 0.2 µm filter (Filtropur S, Sarstedt) and concentrated in Vivaspin 2 columns (Sartorius) for 30 minutes at 4000 rpm (1915x g) and 4 °C. Protein concentration was measured with Pierce BCA Protein Assay (Thermo Fisher Scientific).

Two biological replicates of strains DE9686, α16 and α528 with three technical replicates each were analyzed by LC-MS/MS. Protein samples were digested in solution according to Muntel *et al*.^52^. Resulting digests were loaded on 20 cm self packed columns attached to a Proxeon Easy nLC (Thermo Fisher Scientific) and washed/desalted with a loading volume of 10 μL at a flow of 700 nL/min and a maximum pressure of 220 bar. Separation of peptides was achieved by application of a binary non-linear 150 min gradient from 5–50% acetonitrile in 0.1% acetic acid. The self-packed columns were mounted in a modified nanoelectrospray ion source with liquid junction of the voltage (2400 V) applied between the orifice and the emitter tip. MS and MS/MS data were acquired with the LTQ-Velos Orbitrap mass spectrometer (Thermo Fisher Scientific). After a survey scan in the Orbitrap (R = 30000) MS/MS data were recorded for the twenty most intensive precursor ions in the linear ion trap. Singly charged ions were not taken into account for MS/MS analysis. The lock mass option was enabled throughout the analysis.

### Label-free quantification with Rosetta Elucidator

The replicates were run as described and*.raw- data were subsequently loaded into the Elucidator system (Rosetta Biosoftware). Here, for differential label free quantification between the three strains, the LC- MS/MS runs were first aligned, intensity scaled and features were detected as described previously^52^. Feature intensities were later used for quantification of peptides and rolled up for proteins after database searching. MS/MS data were extracted and subjected to database searching using the Sorcerer Sequest version 4.0.3 (rev. 11) (SageN Research) against a *N. meningitidis* target-decoy protein sequence database (complete proteome set of *N. meningitidis* α710 or MC58) with a set of common laboratory contaminants). The searches were done using the following parameters: enzyme type, trypsin (KR); peptide tolerance, 10 ppm; tolerance for fragment ions, 1 amu; b- and y-ion series; variable modification, methionine (15.99 Da). The search results were imported and statistically analyzed within the Elucidator system by the Peptide Teller leading to a maximum false positive rate of 1% on peptide level. Results for protein intensity data were only taken for further analysis with results for two or more peptides.

The mass spectrometry proteomics data have been deposited to the ProteomeXchange Consortium via the PRIDE^53^ partner repository with the dataset identifier PXD010505

### Screening for NspA phase-variation

Strain WUE4973 (DE9686ΔcsbΔfHbpΔmutS, ‘5As’-nspA promoter) at 10^7^ CFU/ml was incubated in VBS/BSA containing 15% NHS for 30 minutes at 37 °C. Serial dilutions were plated on blood agar plates to verify reduction of CFU by factor 10,000 by serum treatment. Recovered bacteria underwent a total of three cycles of serum stress to select for phase variants with increased serum resistance (*e.g*. by phase variation due to switching from ‘5A’ to ‘6A’ in the promoter of NspA). Grown colonies in each serum stress cycle were transferred onto nitrocellulose membrane, heat-inactivated and probed with monoclonal antibodies against NspA and OpcA. A total of 100 clones from the third cycle of serum stress were chosen for PCR amplification of the NspA promoter region using primers KH145 and KH146 and the PCR products were sequenced to analyze the 5/6 A-tract as readout for phase variation.

### NspA ELISA

The NspA ELISA was performed with modifications according to a published protocol^26^. *N. meningitidis* grown on GC chocolate agar overnight at 37 °C with 5% CO_2_ were adjusted to an optical density at 600 nm (OD_600_) of 0.1 in PBS and heat-inactivated for 30 minutes at 56 °C. Per well, 50 µl of the bacterial suspensions were let to dry at room temperature in 96-well flat bottom tissue culture plates (Sarstedt) in duplicates. Plates were washed once (PBS/0.1% Tween-20) and incubated with 100 µl blocking buffer (PBS/2% skimmed milk) for 1 h at 37 °C. Then, 50 µl of mAb clone 14C7 diluted 1:100 in blocking buffer were added and incubated at 4 °C overnight^32^. ELISA plates were washed and 50 µl peroxidase-conjugated AffiniPure Goat Anti-Mouse IgG + IgM (H+L) (Jackson ImmunoResearch) diluted in PBS/0.1% Tween-20/1% BSA were added to the wells for 1 h at room temperature. After washing ABTS substrate (1 mg/ml; Sigma-Aldrich) was added until colorimetric reaction was visible and then OD_414_ was measured. The ELISA was performed in four instances for all strains and OD_414_ values normalized based on the measurement of two control strains spotted on each individual plate.

### nspA DNA sequence analysis

PCR was performed on boiled bacterial suspension of *N. meningitidis* strains listed in Supplementary Table S2 using the primers nspA1 and nspA2 (see also Supplementary Table S5). PCR products were sequenced according to standard protocols.

### RNA isolation and Quantitative real-time PCR (qRT-PCR)

Meningococcal strains were grown on GC chocolate agar overnight at 37 °C and 5% CO_2_ and suspended in 1xPBS at OD_600_ 0.5. Of this suspension, 500 µl were directly added to 1 ml RNAprotect Bacteria Reagent (QIAGEN). RNA isolation was carried out using the RNeasy Mini Kit (QIAGEN). Completeness of DNA digestion with RNase-free recombinant DNase I was verified by PCR. RNA integrity was verified on a Agilent 2100 Bioanalyzer. Reverse transcription was done with SuperscriptII (Invitrogen). qRT-PCR analysis was performed using the StepOnePlus™ system (Thermo Fisher Scientific). Primers were designed using the Primer Express 3.0 software (Thermo Fisher Scientific). Primer efficiency and unique melting temperature were tested using relative standard curve experiments. qRT-PCR reactions comprised the 2x Power SYBR Green Master Mix, 15 ng cDNA and primers (see Table S5) at a final concentration of 900 nM. Negative controls without cDNA template were included in each run. RQ values (relative quantity) representing the fold change of expression of the investigated gene are provided as means of two RNA isolations from independent bacterial cultures with two RT experiments each. Data analysis was done with the StepOnePlus™ software based on the ΔΔCT method.

### Whole cell fH binding assay

Meningococcal strains and mutants were grown overnight on chocolate agar plates supplemented with the appropriate antibiotics (erythromycin or spectinomycin) and incubated as stated above. Overnight cultures were resuspended in 1xPBS, adjusted to OD_600_ 1.0 and heat-inactivated for 30 min at 65 °C. Suspensions were diluted to OD_600_ 0.1 and 50 µl per well thereof were dried at room temperature in 96-well plates. Plates were washed thrice (1xPBS/0.05% Tween-20) and subsequently blocked with 1xPBS/5% overnight. After incubation with 50 µl of 5% NHS (in 1xPBS/1% BSA) for 1 h, the plate was washed thrice. Then, 50 µl of goat-anti-human fH (Complement Technologies) in 1:2000 dilution in 1xPBS/1% BSA were added and incubated for 1 h. After three washes with PBS/0.05% Tween-20, 50 µl donkey-anti-goat-HRP conjugate (1:10,000 in PBS/1% BSA) were added for 1 h before washing thrice again. Colorimetric detection was done by addition of TMB substrate (ThermoFisher) and stopped with 1 volume of 1 M sulfuric acid and OD_450_ was measured on an ELISA reader.

### Whole cell ELISA for capsule detection

ELISA plates were prepared as described for the fH binding assay above. After three washes (1xPBS/0.05% Tween-20), blocking was done with 5% BSA in 1xPBS overnight and 50 µl primary antibody (anti-serogroup B mouse monocolonal antibody mAb735^54^) diluted to 800 ng/µl in 1xPBS/1% BSA was added per well for 1 h. After three washes with PBS/0.05% Tween-20, 50 µl of goat-anti-mouse-HRP (Jackson ImmunoResearch) diluted 1:10,000 in 1xPBS/1% BSA was added for 1 h. After three washes (1xPBS/0.05% Tween-20), the colorimetric reaction was started by adding 100 µl of TMB substrate (ThermoFisher) per well and stopped by addition of 100 µl 1 M sulfuric acid before reading OD_450_ on an ELISA reader.

## Supplementary information

supplementary data

## Data Availability

All data and materials associated with this publication are available upon request. The mass spectrometry proteomics data have been deposited to the ProteomeXchange Consortium via the PRIDE^53^ partner repository with the dataset identifier PXD010505.
