# Supplementary material for: A homopolymeric adenosine tract in the promoter region of nspA influences factor H-mediated serum resistance in Neisseria meningitidis
Source: Sci Rep. 2019 Feb 25;9:2736. doi: 10.1038/s41598-019-39231-0 (PMC6389960; doi:10.1038/s41598-019-39231-0)
Supplement: Supplementary file 1 — supplementary data [file 41598_2019_39231_MOESM1_ESM.pdf]

Supplementary information to

A homopolymeric adenosine tract in the promoter region of *nspA* influences factor H-mediated serum resistance in *Neisseria meningitidis*

Heike Claus[a], Kerstin Hubert[a], Dörte Becher[b], Andreas Otto[b], Marie-Christin Pawlik[a], Ines Lappann[a], Lea Strobel[a], Ulrich Vogel[a] and Kay Johswich[a]

[a] Institute for Hygiene and Microbiology, University of Würzburg, Germany

[b] Institute for Microbiology, University of Greifswald, Germany

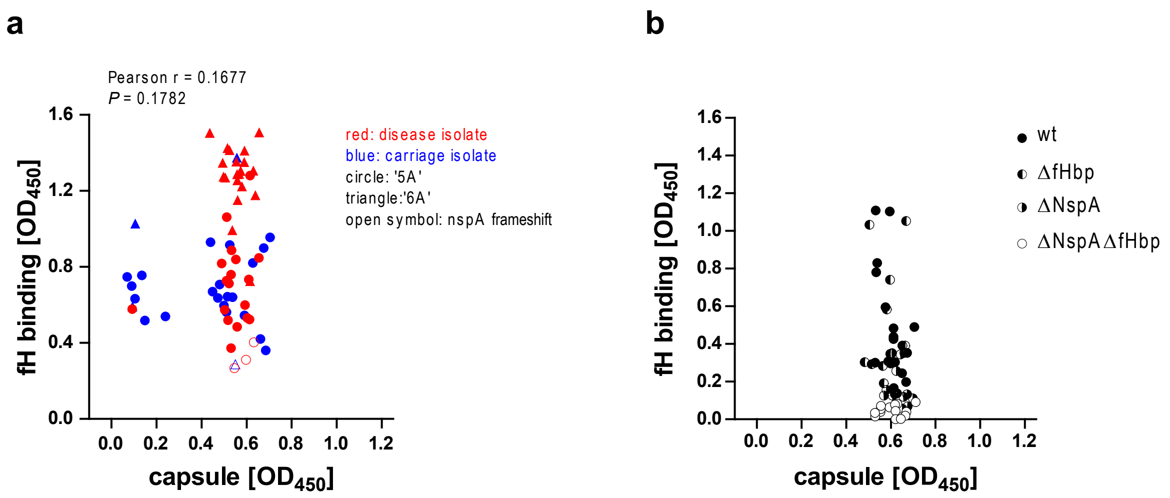

Figure S1: fH-binding to strains in Figure 5 versus their capsulation status. (A) fH-binding to cc41/44 strains as assessed in Fig. 5b plotted against their capsulation as measured by whole cell ELISA. Results of Pearson's correlation analysis is plotted above graph. **(B)** fH-binding to cc41/44 strains and their correponding mutants lacking fHbp, NspA, or both, as assessed in Fig. 5c against their capsulation as determined by whole cell ELISA. Note that all 'wt' strains from the three panels of Fig.5c have been pooled here, irrespective of their '6A' or '5A' status or disease phenotype; the same applies to the mutants. Evidently, all strains in Fig.5c showed a similar degree of capsulation.

supplementary table S1: Results of proteomic analysis

comparison strain DE9686 vesus α528

<table>
<tr><th></th><th>Replicate 1</th><th>Replicate 1</th><th>Replicate 2</th><th>Replicate 2</th><th></th><th></th></tr>
<tr><th>Protein ID</th><th>Log (DE9686/a528)</th><th>neg. log10 of P-value (Bonferroni-adjusted)</th><th>Log (DE9686/a528)</th><th>neg. log10 of P-value (Bonferroni-adjusted)</th><th>Protein Code</th><th>Protein Description</th></tr>
<tr><td>118148</td><td>1,409</td><td>18,254</td><td>0,789</td><td>2,191</td><td>GI|308389936|GB|ADO32256.1|</td><td>putative isoleucyl-tRNA synthetase [Neisseria meningitidis alpha710]</td></tr>
<tr><td>117484</td><td>1,384</td><td>40,296</td><td>1,140</td><td>1,901</td><td>GI|308389123|GB|ADO31443.1|</td><td>glutamate dehydrogenase, NAD-specific [Neisseria meningitidis alpha710]</td></tr>
<tr><td>116028</td><td>1,161</td><td>40,296</td><td>0,715</td><td>6,135</td><td>GI|7225888|GB|AAF41081.1|</td><td>outer membrane protein NspA [Neisseria meningitidis MC58]</td></tr>
<tr><td>116675</td><td>0,641</td><td>18,847</td><td>0,691</td><td>10,222</td><td>GI|308389915|GB|ADO32235.1|</td><td>riboflavin biosynthesis protein RibD [Neisseria meningitidis alpha710]</td></tr>
<tr><td>117400</td><td>0,438</td><td>10,838</td><td>0,377</td><td>2,598</td><td>GI|308389308|GB|ADO31628.1|</td><td>bacterioferritin B [Neisseria meningitidis MC58]</td></tr>
<tr><td>116671</td><td>-0,113</td><td>1,050</td><td>-0,318</td><td>3,161</td><td>GI|308390235|GB|ADO32555.1|</td><td>electron transfer flavoprotein beta-subunit [Neisseria meningitidis alpha710]</td></tr>
<tr><td>117180</td><td>-0,147</td><td>3,915</td><td>-0,162</td><td>1,675</td><td>GI|7225862|GB|AAF41057.1|</td><td>iron(III) ABC transporter, periplasmic binding protein [Neisseria meningitidis MC58]</td></tr>
<tr><td>116195</td><td>-0,168</td><td>8,014</td><td>-0,430</td><td>1,610</td><td>GI|308388373|GB|ADO30693.1|</td><td>50S ribosomal protein L6 [Neisseria meningitidis alpha710]</td></tr>
<tr><td>116013</td><td>-0,190</td><td>2,621</td><td>-0,417</td><td>0,928</td><td>GI|308388368|GB|ADO30688.1|</td><td>50S ribosomal protein L14 [Neisseria meningitidis MC58]</td></tr>
<tr><td>116712</td><td>-0,243</td><td>1,549</td><td>-0,686</td><td>5,892</td><td>GI|7226294|GB|AAF41451.1|</td><td>class 5 outer membrane protein [Neisseria meningitidis MC58]</td></tr>
<tr><td>116034</td><td>-0,332</td><td>29,721</td><td>-0,280</td><td>4,228</td><td>GI|308388349|GB|ADO30669.1|</td><td>DNA-directed RNA polymerase beta chain [Neisseria meningitidis alpha710]</td></tr>
<tr><td>117989</td><td>-0,367</td><td>40,296</td><td>-0,456</td><td>5,735</td><td>GI|308388377|GB|ADO30697.1|</td><td>50S ribosomal protein L15 [Neisseria meningitidis MC58]</td></tr>
<tr><td>116458</td><td>-0,382</td><td>1,677</td><td>-0,564</td><td>3,928</td><td>GI|308389350|GB|ADO31670.1|</td><td>putative bifunctional 3,4-dihydroxy-2-butanone 4- phosphate synthase/GTP cyclohydrolase II [Neisseria meningitidis alpha710]</td></tr>
<tr><td>116776</td><td>-0,451</td><td>40,296</td><td>-0,620</td><td>3,269</td><td>GI|7226680|GB|AAF41800.1|</td><td>phosphoribosylaminoimidazole carboxylase, catalytic subunit [Neisseria meningitidis MC58]</td></tr>
<tr><td>116951</td><td>-0,482</td><td>0,967</td><td>-0,430</td><td>2,338</td><td>GI|308388962|GB|ADO31282.1|</td><td>hypothetical protein NMBB_0876 [Neisseria meningitidis alpha710]</td></tr>
<tr><td>116330</td><td>-0,592</td><td>40,296</td><td>-0,627</td><td>29,100</td><td>GI|308388235|GB|ADO30555.1|</td><td>phosphoglycerate kinase [Neisseria meningitidis alpha710]</td></tr>
<tr><td>117092</td><td>-0,614</td><td>7,473</td><td>-0,349</td><td>7,024</td><td>GI|308389533|GB|ADO31853.1|</td><td>porin, class I outer membrane protein [Neisseria meningitidis alpha710]</td></tr>
<tr><td>118281</td><td>-0,792</td><td>4,794</td><td>-1,521</td><td>17,851</td><td>GI|7227105|GB|AAF42183.1|</td><td>carbamoyl-phosphate synthase, small subunit [Neisseria meningitidis MC58]</td></tr>
<tr><td>115926</td><td>-1,223</td><td>18,800</td><td>-0,758</td><td>29,100</td><td>GI|7225438|GB|AAF40672.1|</td><td>catalase [Neisseria meningitidis MC58]</td></tr>
</table>

comparison strain DE9686 vesus α16

<table>
<tr><th></th><th>Replicate 1</th><th>Replicate 1</th><th>Replicate 2</th><th>Replicate 2</th><th></th><th></th></tr>
<tr><th>Protein ID</th><th>Log (DE9686/a16)</th><th>neg. log10 of P-value (Bonferroni-adjusted)</th><th>Log (DE9686/a16)</th><th>neg. log10 of P-value (Bonferroni-adjusted)</th><th>Protein Code</th><th>Protein Description</th></tr>
<tr><td>117805</td><td>2,077</td><td>41,556</td><td>1,349</td><td>0,893</td><td>GI|308389246|GB|ADO31566.1|</td><td>conserved hypothetical protein [Neisseria meningitidis MC58]</td></tr>
<tr><td>116834</td><td>1,757</td><td>13,808</td><td>1,449</td><td>9,416</td><td>GI|308389239|GB|ADO31559.1|</td><td>hypothetical protein NMB1084 [Neisseria meningitidis MC58]</td></tr>
<tr><td>117052</td><td>1,484</td><td>41,556</td><td>1,534</td><td>2,196</td><td>GI|7227228|GB|AAF42297.1|</td><td>aldehyde dehydrogenase A [Neisseria meningitidis MC58]</td></tr>
<tr><td>116028</td><td>1,359</td><td>41,556</td><td>0,975</td><td>8,645</td><td>GI|7225888|GB|AAF41081.1|</td><td>outer membrane protein NspA [Neisseria meningitidis MC58]</td></tr>
<tr><td>118171</td><td>0,702</td><td>19,509</td><td>0,600</td><td>1,658</td><td>GI|REV_7226789|GB|AAF41898.1|</td><td>conserved hypothetical protein [Neisseria meningitidis MC58]</td></tr>
<tr><td>117503</td><td>0,324</td><td>1,804</td><td>0,485</td><td>1,290</td><td>GI|308389367|GB|ADO31687.1|</td><td>hypothetical protein NMB1035 [Neisseria meningitidis MC58]</td></tr>
<tr><td>115869</td><td>0,236</td><td>1,161</td><td>0,543</td><td>1,704</td><td>GI|308389706|GB|ADO32026.1|</td><td>aconitate hydratase [Neisseria meningitidis alpha710]</td></tr>
<tr><td>116426</td><td>0,216</td><td>17,167</td><td>-0,283</td><td>2,391</td><td>GI|308388938|GB|ADO31258.1|</td><td>host factor-I [Neisseria meningitidis alpha710]</td></tr>
<tr><td>117568</td><td>0,141</td><td>3,214</td><td>-0,474</td><td>3,189</td><td>GI|308389782|GB|ADO32102.1|</td><td>DNA-directed RNA polymerase, omega subunit [Neisseria meningitidis MC58]</td></tr>
<tr><td>116672</td><td>-0,048</td><td>1,742</td><td>-0,494</td><td>4,839</td><td>GI|308389950|GB|ADO32270.1|</td><td>carbamoyl phosphate synthase small subunit [Neisseria meningitidis alpha710]</td></tr>
<tr><td>118429</td><td>-0,051</td><td>1,471</td><td>-0,341</td><td>2,915</td><td>GI|7226284|GB|AAF41442.1|</td><td>ferredoxin--NADP reductase [Neisseria meningitidis MC58]</td></tr>
<tr><td>116556</td><td>-0,065</td><td>1,006</td><td>-0,385</td><td>2,232</td><td>GI|308389136|GB|ADO31456.1|</td><td>single-strand binding protein [Neisseria meningitidis MC58]</td></tr>
<tr><td>116219</td><td>-0,076</td><td>3,621</td><td>-0,297</td><td>2,837</td><td>GI|308388370|GB|ADO30690.1|</td><td>50S ribosomal protein L5 [Neisseria meningitidis MC58]</td></tr>
<tr><td>116996</td><td>-0,079</td><td>2,299</td><td>-0,585</td><td>12,950</td><td>GI|7226630|GB|AAF41756.1|</td><td>glucose-6-phosphate 1-dehydrogenase [Neisseria meningitidis MC58]</td></tr>
<tr><td>117026</td><td>-0,083</td><td>7,406</td><td>-0,548</td><td>7,568</td><td>GI|308389908|GB|ADO32228.1|</td><td>pilO protein [Neisseria meningitidis MC58]</td></tr>
<tr><td>116504</td><td>-0,094</td><td>10,038</td><td>-0,497</td><td>3,337</td><td>GI|7225545|GB|AAF40769.1|</td><td>50S ribosomal protein L27 [Neisseria meningitidis MC58]</td></tr>
<tr><td>116601</td><td>-0,096</td><td>4,621</td><td>-0,408</td><td>9,670</td><td>GI|308389625|GB|ADO31945.1|</td><td>elongation factor P (EF-P) [Neisseria meningitidis MC58]</td></tr>
<tr><td>116042</td><td>-0,101</td><td>0,987</td><td>-0,217</td><td>2,861</td><td>GI|7227385|GB|AAF42437.1|</td><td>argininosuccinate synthase [Neisseria meningitidis MC58]</td></tr>
<tr><td>116469</td><td>-0,102</td><td>4,692</td><td>-0,315</td><td>0,996</td><td>GI|7225321|GB|AAF40565.1|</td><td>aspartate carbamoyltransferase, catalytic subunit [Neisseria meningitidis MC58]</td></tr>
<tr><td>116776</td><td>-0,113</td><td>6,630</td><td>-0,401</td><td>1,545</td><td>GI|7226680|GB|AAF41800.1|</td><td>phosphoribosylaminoimidazole carboxylase, catalytic subunit [Neisseria meningitidis MC58]</td></tr>
<tr><td>115934</td><td>-0,114</td><td>27,936</td><td>-0,368</td><td>1,543</td><td>GI|308390105|GB|ADO32425.1|</td><td>bifunctional ornithine acetyltransferase/N- acetylglutamate synthase protein [Neisseria meningitidis alpha710]</td></tr>
<tr><td>117980</td><td>-0,115</td><td>2,122</td><td>-0,722</td><td>5,525</td><td>GI|308388778|GB|ADO31098.1|</td><td>30S ribosomal protein S16 [Neisseria meningitidis MC58]</td></tr>
<tr><td>116658</td><td>-0,115</td><td>41,556</td><td>-0,245</td><td>6,813</td><td>GI|308389701|GB|ADO32021.1|</td><td>macrophage infectivity potentiator [Neisseria meningitidis MC58]</td></tr>
<tr><td>116038</td><td>-0,118</td><td>4,471</td><td>-0,119</td><td>2,781</td><td>GI|308389651|GB|ADO31971.1|</td><td>nicotinic acid phosphoribosyltransferase [Neisseria meningitidis alpha710]</td></tr>
<tr><td>115941</td><td>-0,120</td><td>2,084</td><td>-0,427</td><td>3,672</td><td>GI|308388950|GB|ADO31270.1|</td><td>cysteine synthase [Neisseria meningitidis MC58]</td></tr>
<tr><td>116751</td><td>-0,122</td><td>1,502</td><td>-0,333</td><td>7,950</td><td>GI|7226632|GB|AAF41757.1|</td><td>phosphogluconate dehydratase [Neisseria meningitidis MC58]</td></tr>
<tr><td>116501</td><td>-0,135</td><td>22,284</td><td>-0,572</td><td>5,092</td><td>GI|308388344|GB|ADO30664.1|</td><td>transcription antitermination protein NusG [Neisseria meningitidis MC58]</td></tr>
<tr><td>116363</td><td>-0,136</td><td>2,918</td><td>-0,479</td><td>7,750</td><td>GI|308389475|GB|ADO31795.1|</td><td>aspartyl/glutamyl-tRNA amidotransferase subunit B [Neisseria meningitidis alpha710]</td></tr>
<tr><td>116810</td><td>-0,137</td><td>15,143</td><td>-0,544</td><td>4,304</td><td>GI|308389508|GB|ADO31828.1|</td><td>oxidoreductase, Sol/DevB family [Neisseria meningitidis MC58]</td></tr>
<tr><td>116058</td><td>-0,138</td><td>2,840</td><td>-0,497</td><td>8,582</td><td>GI|7227088|GB|AAF42168.1|</td><td>isoleucyl-tRNA synthetase [Neisseria meningitidis MC58]</td></tr>
<tr><td>115969</td><td>-0,144</td><td>5,270</td><td>-0,340</td><td>2,232</td><td>GI|308388354|GB|ADO30674.1|</td><td>30S ribosomal protein S7 [Neisseria meningitidis MC58]</td></tr>
<tr><td>116693</td><td>-0,150</td><td>15,747</td><td>-0,563</td><td>11,413</td><td>GI|308388663|GB|ADO30983.1|</td><td>putative spermidine/putrescine transport system substrate-binding protein [Neisseria meningitidis alpha710]</td></tr>
<tr><td>116913</td><td>-0,158</td><td>7,816</td><td>-0,711</td><td>6,297</td><td>GI|308388385|GB|ADO30705.1|</td><td>50S ribosomal protein L17 [Neisseria meningitidis alpha710]</td></tr>
<tr><td>115905</td><td>-0,164</td><td>2,991</td><td>-0,468</td><td>6,700</td><td>GI|308388267|GB|ADO30587.1|</td><td>peptide methionine sulfoxide reductase [Neisseria meningitidis MC58]</td></tr>
<tr><td>117046</td><td>-0,164</td><td>24,837</td><td>-0,552</td><td>12,765</td><td>GI|7226669|GB|AAF41790.1|</td><td>outer membrane protein PorA [Neisseria meningitidis MC58]</td></tr>
<tr><td>116452</td><td>-0,171</td><td>2,206</td><td>-0,558</td><td>0,997</td><td>GI|308389657|GB|ADO31977.1|</td><td>YgbB/YacN family protein [Neisseria meningitidis MC58]</td></tr>
<tr><td>116806</td><td>-0,174</td><td>3,428</td><td>-0,720</td><td>11,530</td><td>GI|308388229|GB|ADO30549.1|</td><td>putative oxidoreductase [Neisseria meningitidis alpha710]</td></tr>
<tr><td>117612</td><td>-0,174</td><td>4,167</td><td>-0,514</td><td>3,645</td><td>GI|7225950|GB|AAF41136.1|</td><td>50S ribosomal protein L20 [Neisseria meningitidis MC58]</td></tr>
<tr><td>116166</td><td>-0,178</td><td>3,874</td><td>-0,373</td><td>0,940</td><td>GI|308388441|GB|ADO30761.1|</td><td>acyl carrier protein [Neisseria meningitidis MC58]</td></tr>
<tr><td>118271</td><td>-0,180</td><td>0,989</td><td>-0,547</td><td>6,787</td><td>GI|308388664|GB|ADO30984.1|</td><td>30S ribosomal protein S20 [Neisseria meningitidis MC58]</td></tr>
<tr><td>118110</td><td>-0,186</td><td>17,148</td><td>-0,319</td><td>1,027</td><td>GI|308388369|GB|ADO30689.1|</td><td>50S ribosomal protein L24 [Neisseria meningitidis MC58]</td></tr>
<tr><td>117621</td><td>-0,197</td><td>6,521</td><td>-0,995</td><td>5,473</td><td>GI|308388367|GB|ADO30687.1|</td><td>30S ribosomal protein S17 [Neisseria meningitidis MC58]</td></tr>
<tr><td>117886</td><td>-0,206</td><td>1,203</td><td>-0,592</td><td>3,734</td><td>GI|308388360|GB|ADO30680.1|</td><td>50S ribosomal protein L23 [Neisseria meningitidis alpha710]</td></tr>
<tr><td>116188</td><td>-0,208</td><td>24,228</td><td>-0,695</td><td>7,778</td><td>GI|308389906|GB|ADO32226.1|</td><td>pilM protein [Neisseria meningitidis MC58]</td></tr>
<tr><td>116495</td><td>-0,208</td><td>10,036</td><td>-0,871</td><td>2,342</td><td>GI|7225831|GB|AAF41029.1|</td><td>hitA protein [Neisseria meningitidis MC58]</td></tr>
<tr><td>115889</td><td>-0,212</td><td>26,851</td><td>-0,345</td><td>4,226</td><td>GI|308390239|GB|ADO32559.1|</td><td>glyceraldehyde 3-phosphate dehydrogenase C [Neisseria meningitidis alpha710]</td></tr>
<tr><td>117675</td><td>-0,215</td><td>17,375</td><td>-0,545</td><td>4,447</td><td>GI|308388513|GB|ADO30833.1|</td><td>putative thiol:disulfide interchange protein [Neisseria meningitidis alpha710]</td></tr>
<tr><td>116516</td><td>-0,216</td><td>7,415</td><td>-0,359</td><td>1,428</td><td>GI|308390201|GB|ADO32521.1|</td><td>uridylate kinase [Neisseria meningitidis MC58]</td></tr>
<tr><td>116831</td><td>-0,217</td><td>17,724</td><td>-0,609</td><td>6,862</td><td>GI|7226057|GB|AAF41235.1|</td><td>heat shock protein HtpX [Neisseria meningitidis MC58]</td></tr>
<tr><td>118095</td><td>-0,224</td><td>4,291</td><td>-0,562</td><td>21,451</td><td>GI|308389846|GB|ADO32166.1|</td><td>biopolymer transport protein ExbD [Neisseria meningitidis MC58]</td></tr>
<tr><td>116093</td><td>-0,233</td><td>3,436</td><td>-1,033</td><td>35,272</td><td>GI|308388362|GB|ADO30682.1|</td><td>30S ribosomal protein S19 [Neisseria meningitidis MC58]</td></tr>
<tr><td>117655</td><td>-0,237</td><td>1,473</td><td>-0,736</td><td>5,947</td><td>GI|308388668|GB|ADO30988.1|</td><td>hypothetical protein NMB0467 [Neisseria meningitidis MC58]</td></tr>
<tr><td>117513</td><td>-0,237</td><td>37,580</td><td>-0,560</td><td>6,114</td><td>GI|308389703|GB|ADO32023.1|</td><td>aminopeptidase A [Neisseria meningitidis alpha710]</td></tr>
<tr><td>116651</td><td>-0,242</td><td>41,556</td><td>-0,458</td><td>16,447</td><td>GI|308390137|GB|ADO32457.1|</td><td>porin, major outer membrane protein P.I [Neisseria meningitidis alpha710]</td></tr>
<tr><td>117031</td><td>-0,249</td><td>19,761</td><td>-0,462</td><td>5,486</td><td>GI|308388361|GB|ADO30681.1|</td><td>50S ribosomal protein L2 [Neisseria meningitidis alpha710]</td></tr>
<tr><td>118227</td><td>-0,250</td><td>8,073</td><td>-0,953</td><td>7,321</td><td>GI|REV_308389975|GB|ADO32295.1|</td><td>N-acetylglutamate synthase [Neisseria meningitidis MC58]</td></tr>
<tr><td>116311</td><td>-0,251</td><td>41,556</td><td>-0,555</td><td>7,269</td><td>GI|7227094|GB|AAF42174.1|</td><td>formate--tetrahydrofolate ligase [Neisseria meningitidis MC58]</td></tr>
<tr><td>117179</td><td>-0,259</td><td>7,111</td><td>-0,782</td><td>13,011</td><td>GI|7225819|GB|AAF41017.1|</td><td>50s ribosomal protein L19 [Neisseria meningitidis MC58]</td></tr>
<tr><td>117071</td><td>-0,260</td><td>19,166</td><td>-1,340</td><td>35,272</td><td>GI|308388970|GB|ADO31290.1|</td><td>putative phage shock protein E precursor [Neisseria meningitidis MC58]</td></tr>
<tr><td>116271</td><td>-0,261</td><td>5,687</td><td>-0,356</td><td>1,918</td><td>GI|308388353|GB|ADO30673.1|</td><td>30S ribosomal protein S12 [Neisseria meningitidis MC58]</td></tr>
<tr><td>116957</td><td>-0,268</td><td>41,556</td><td>-0,254</td><td>2,974</td><td>GI|308388422|GB|ADO30742.1|</td><td>dihydrodipicolinate reductase [Neisseria meningitidis alpha710]</td></tr>
<tr><td>116087</td><td>-0,286</td><td>7,345</td><td>-0,506</td><td>13,066</td><td>GI|308388744|GB|ADO31064.1|</td><td>grpE protein [Neisseria meningitidis MC58]</td></tr>
<tr><td>117989</td><td>-0,292</td><td>10,010</td><td>-0,437</td><td>2,342</td><td>GI|308388377|GB|ADO30697.1|</td><td>50S ribosomal protein L15 [Neisseria meningitidis MC58]</td></tr>
<tr><td>116613</td><td>-0,297</td><td>41,556</td><td>-0,444</td><td>16,922</td><td>GI|7226698|GB|AAF41816.1|</td><td>transketolase [Neisseria meningitidis MC58]</td></tr>
<tr><td>116697</td><td>-0,303</td><td>41,556</td><td>-0,275</td><td>6,234</td><td>GI|308388590|GB|ADO30910.1|</td><td>ABC transporter, ATP-binding protein [Neisseria meningitidis MC58]</td></tr>
<tr><td>118127</td><td>-0,304</td><td>41,556</td><td>-0,601</td><td>2,271</td><td>GI|308389139|GB|ADO31459.1|</td><td>transketolase [Neisseria meningitidis alpha710]</td></tr>
<tr><td>116068</td><td>-0,314</td><td>27,596</td><td>-0,726</td><td>35,272</td><td>GI|308389505|GB|ADO31825.1|</td><td>glucose-6-phosphate isomerase [Neisseria meningitidis alpha710]</td></tr>
<tr><td>116941</td><td>-0,315</td><td>7,778</td><td>-0,762</td><td>3,378</td><td>GI|308389056|GB|ADO31376.1|</td><td>putative superoxide dismutase [Neisseria meningitidis alpha710]</td></tr>
<tr><td>116919</td><td>-0,330</td><td>1,909</td><td>-1,027</td><td>1,612</td><td>GI|308389834|GB|ADO32154.1|</td><td>membrane fusion protein [Neisseria meningitidis alpha710]</td></tr>
<tr><td>116871</td><td>-0,333</td><td>8,131</td><td>-0,485</td><td>1,891</td><td>GI|308389903|GB|ADO32223.1|</td><td>cytochrome c4 [Neisseria meningitidis MC58]</td></tr>
<tr><td>116382</td><td>-0,335</td><td>5,623</td><td>-0,404</td><td>15,322</td><td>GI|308388381|GB|ADO30701.1|</td><td>30S ribosomal protein S13 [Neisseria meningitidis MC58]</td></tr>
<tr><td>115872</td><td>-0,342</td><td>41,556</td><td>-0,378</td><td>3,713</td><td>GI|308389798|GB|ADO32118.1|</td><td>glycine dehydrogenase [Neisseria meningitidis alpha710]</td></tr>
<tr><td>116823</td><td>-0,355</td><td>0,952</td><td>-1,099</td><td>5,799</td><td>GI|308389801|GB|ADO32121.1|</td><td>tRNA (uracil-5-)-methyltransferase [Neisseria meningitidis alpha710]</td></tr>
<tr><td>117149</td><td>-0,364</td><td>41,556</td><td>-0,772</td><td>35,272</td><td>GI|7226015|GB|AAF41196.1|</td><td>conserved hypothetical protein [Neisseria meningitidis MC58]</td></tr>
<tr><td>116026</td><td>-0,364</td><td>1,107</td><td>-1,015</td><td>2,243</td><td>GI|308390221|GB|ADO32541.1|</td><td>hypothetical protein NMBB_2436 [Neisseria meningitidis alpha710]</td></tr>
<tr><td>118385</td><td>-0,366</td><td>0,996</td><td>-0,655</td><td>2,910</td><td>GI|308390040|GB|ADO32360.1|</td><td>hypothetical protein NMBB_2218 [Neisseria meningitidis alpha710]</td></tr>
<tr><td>117590</td><td>-0,373</td><td>2,333</td><td>-0,447</td><td>1,445</td><td>GI|308390028|GB|ADO32348.1|</td><td>putative glycyl-tRNA synthetase beta subunit [Neisseria meningitidis alpha710]</td></tr>
<tr><td>117000</td><td>-0,404</td><td>4,029</td><td>-0,774</td><td>29,850</td><td>GI|308390048|GB|ADO32368.1|</td><td>30S ribosomal protein S21 [Neisseria meningitidis MC58]</td></tr>
<tr><td>117218</td><td>-0,490</td><td>29,624</td><td>-0,739</td><td>13,340</td><td>GI|308388961|GB|ADO31281.1|</td><td>putative uracil phosphoribosyltransferase [Neisseria meningitidis alpha710]</td></tr>
<tr><td>117768</td><td>-0,504</td><td>22,985</td><td>-1,046</td><td>22,249</td><td>GI|308388382|GB|ADO30702.1|</td><td>30S ribosomal protein S11 [Neisseria meningitidis MC58]</td></tr>
<tr><td>116951</td><td>-0,510</td><td>6,225</td><td>-0,642</td><td>13,550</td><td>GI|308388962|GB|ADO31282.1|</td><td>hypothetical protein NMBB_0876 [Neisseria meningitidis alpha710]</td></tr>
<tr><td>116458</td><td>-0,513</td><td>41,556</td><td>-0,929</td><td>25,889</td><td>GI|308389350|GB|ADO31670.1|</td><td>putative bifunctional 3,4-dihydroxy-2-butanone 4- phosphate synthase/GTP cyclohydrolase II [Neisseria meningitidis alpha710]</td></tr>
<tr><td>117047</td><td>-0,573</td><td>23,743</td><td>-0,467</td><td>2,790</td><td>GI|7225870|GB|AAF41064.1|</td><td>inorganic pyrophosphatase [Neisseria meningitidis MC58]</td></tr>
<tr><td>117727</td><td>-0,658</td><td>6,174</td><td>-1,395</td><td>10,802</td><td>GI|308388761|GB|ADO31081.1|</td><td>glycine cleavage system H protein [Neisseria meningitidis MC58]</td></tr>
<tr><td>116330</td><td>-0,672</td><td>41,556</td><td>-0,796</td><td>35,272</td><td>GI|308388235|GB|ADO30555.1|</td><td>phosphoglycerate kinase [Neisseria meningitidis alpha710]</td></tr>
<tr><td>116848</td><td>-0,805</td><td>41,556</td><td>-1,138</td><td>13,860</td><td>GI|7225686|GB|AAF40897.1|</td><td>transferrin-binding protein 2 [Neisseria meningitidis MC58]</td></tr>
<tr><td>118181</td><td>-0,831</td><td>34,386</td><td>-0,497</td><td>2,211</td><td>GI|308388380|GB|ADO30700.1|</td><td>50S ribosomal protein L36 [Neisseria meningitidis MC58]</td></tr>
<tr><td>117529</td><td>-0,839</td><td>41,556</td><td>-0,884</td><td>8,942</td><td>GI|7225378|GB|AAF40618.1|</td><td>50S ribosomal protein L30 [Neisseria meningitidis MC58]</td></tr>
<tr><td>117715</td><td>-0,941</td><td>41,556</td><td>-1,250</td><td>1,789</td><td>GI|308389866|GB|ADO32186.1|</td><td>VapD-related protein [Neisseria meningitidis MC58]</td></tr>
<tr><td>116222</td><td>-1,068</td><td>41,556</td><td>-1,336</td><td>35,272</td><td>GI|7226617|GB|AAF41745.1|</td><td>acetylornithine aminotransferase [Neisseria meningitidis MC58]</td></tr>
<tr><td>118091</td><td>-1,074</td><td>41,556</td><td>-0,838</td><td>1,823</td><td>GI|308389277|GB|ADO31597.1|</td><td>hypothetical protein NMBB_1258 [Neisseria meningitidis alpha710]</td></tr>
<tr><td>116509</td><td>-1,134</td><td>5,148</td><td>-1,416</td><td>3,363</td><td>GI|7226370|GB|AAF41521.1|</td><td>conserved hypothetical protein / ankyrin-related protein [Neisseria meningitidis MC58]</td></tr>
<tr><td>118174</td><td>-1,547</td><td>41,556</td><td>-1,955</td><td>6,513</td><td>GI|7226612|GB|AAF41740.1|</td><td>thioredoxin [Neisseria meningitidis MC58]</td></tr>
</table>

Supplementary table S2: Overview of carriage strains and disease strains used in this study

overview carriage strains (alpha-strains from Bavarian carriage study*)

*Claus H, Maiden MC, Maag R, Frosch M, Vogel U. 2002. Many carried meningococci lack the genes required for capsule synthesis and transport. Microbiology 148:1813-9.

strain (alpha)	sequence type (ST)	No of 'A' in poly-A stretch	clonal complex	serogroup (genotype)	PorA (VR1)	PorA (VR2)	fetA	sequence poly-A stretch in *nspA* promoter	NspA-ELISA (OD)
16	41	5	ST-41/44 complex/Lineage 3	B	7-2	4	1-5	TGC - AAAAAGAAAATTT	0,370
19	44	5	ST-41/44 complex/Lineage 3	B				TGC - AAAAAGAAAATTT	0,292
25	180	5	ST-41/44 complex/Lineage 3	B				TGC - AAAAAGAAAATTT	
27	43	5	ST-41/44 complex/Lineage 3	B				TGC - AAAAAGAAAATTT	
38	44	5	ST-41/44 complex/Lineage 3	B				TGC - AAAAAGAAAATTT	0,318
50	44	5	ST-41/44 complex/Lineage 3	B				TGC - AAAAAGAAAATTT	0,241
56	877	5	ST-41/44 complex/Lineage 3	B				TGC - AAAAAGAAAATTT	
78	44	5	ST-41/44 complex/Lineage 3	B				TGC - AAAAAGAAAATTT	0,301
80	41	5	ST-41/44 complex/Lineage 3	B				TGC - AAAAAGAAAATTT	0,382
84	777	5	ST-41/44 complex/Lineage 3	B				TGC - AAAAAGAAAATTT	
92	136	5	ST-41/44 complex/Lineage 3	B				TGC - AAAAAGAAAATTT	
97	782	5	ST-41/44 complex/Lineage 3	B				TGC - AAAAAGAAAATTT	
100	886	5	ST-41/44 complex/Lineage 3	B				TGC - AAAAAGAAAATTT	
103	1103	5	ST-41/44 complex/Lineage 3	B				TGC - AAAAAGAAAATTT	
108	891	5	ST-41/44 complex/Lineage 3	B				TGC - AAAAAGAAAATTT	
110	1139	5	ST-41/44 complex/Lineage 3	B				TGC - AAAAAGAAAATTT	
116	1126	5	ST-41/44 complex/Lineage 3	B				TGC - AAAAAGAAAATTT	
118	887	5	ST-41/44 complex/Lineage 3	B				TGC - AAAAAGAAAATTT	
128	921	5	ST-41/44 complex/Lineage 3	B				TGC - AAAAAGAAAATTT	
142	877	5	ST-41/44 complex/Lineage 3	B				TGC - AAAAAGAAAATTT	
154	43	5	ST-41/44 complex/Lineage 3	B				TGC - AAAAAGAAAATTT	
154	43	5	ST-41/44 complex/Lineage 3	B				TGC - AAAAAGAAAATTT	
171	833	5	ST-41/44 complex/Lineage 3	B				TGC - AAAAAGAAAATTT	
190	757	5	ST-41/44 complex/Lineage 3	B				TGC - AAAAAGAAAATTT	
207	477	5	ST-41/44 complex/Lineage 3	C				TGC - AAAAAGAAAATTT	0,566
209	833	5	ST-41/44 complex/Lineage 3	B				TGC - AAAAAGAAAATTT	
211	318	5	ST-41/44 complex/Lineage 3	B				TGC - AAAAAGAAAATTT	
219	44	5	ST-41/44 complex/Lineage 3	B				TGC - AAAAAGAAAATTT	0,409
238	136	5	ST-41/44 complex/Lineage 3	B				TGC - AAAAAGAAAATTT	
241	136	5	ST-41/44 complex/Lineage 3	B				TGC - AAAAAGAAAATTT	
250	878	5	ST-41/44 complex/Lineage 3	B				TGC - AAAAAGAAAATTT	
253	41	5	ST-41/44 complex/Lineage 3	B				TGC - AAAAAGAAAATTT	0,402
270	191	5	ST-41/44 complex/Lineage 3	B				TGC - AAAAAGAAAATTT	
280	906	5	ST-41/44 complex/Lineage 3	B				TGC - AAAAAGAAAATTT	
283	877	5	ST-41/44 complex/Lineage 3	B				TGC - AAAAAGAAAATTT	0,452
285	112	5	ST-41/44 complex/Lineage 3	B				TGC - AAAAAGAAAATTT	
287	944	5	ST-41/44 complex/Lineage 3	B				TGC - AAAAAGAAAATTT	
297	337	5	ST-41/44 complex/Lineage 3	C				TGC - AAAAAGAAAATTT	
302	180	5	ST-41/44 complex/Lineage 3	C				TGC - AAAAAGAAAATTT	
311	883	5	ST-41/44 complex/Lineage 3	C				TGC - AAAAAGAAAATTT	
313	921	5	ST-41/44 complex/Lineage 3	B				TGC - AAAAAGAAAATTT	
314	788	5	ST-41/44 complex/Lineage 3	B				TGC - AAAAAGAAAATTT	
317	437	5	ST-41/44 complex/Lineage 3	B				TGC - AAAAAGAAAATTT	
326	112	5	ST-41/44 complex/Lineage 3	B				TGC - AAAAAGAAAATTT	
354	838	5	ST-41/44 complex/Lineage 3	B				TGC - AAAAAGAAAATTT	
379	835	5	ST-41/44 complex/Lineage 3	B				TGC - AAAAAGAAAATTT	
384	835	5	ST-41/44 complex/Lineage 3	B				TGC - AAAAAGAAAATTT	
393	482	5	ST-41/44 complex/Lineage 3	B				TGC - AAAAAGAAAATTT	0,485
401	973	5	ST-41/44 complex/Lineage 3	B				TGC - AAAAAGAAAATTT	
406	1108	5	ST-41/44 complex/Lineage 3	B				TGC - AAAAAGAAAATTT	
415	136	5	ST-41/44 complex/Lineage 3	B				TGC - AAAAAGAAAATTT	
421	112	5	ST-41/44 complex/Lineage 3	B				TGC - AAAAAGAAAATTT	
436	835	5	ST-41/44 complex/Lineage 3	B				TGC - AAAAAGAAAATTT	
440	170	5	ST-41/44 complex/Lineage 3	B				TGC - AAAAAGAAAATTT	
455	44	5	ST-41/44 complex/Lineage 3	B				TGC - AAAAAGAAAATTT	0,253
456	110	5	ST-41/44 complex/Lineage 3	B				TGC - AAAAAGAAAATTT	
470	437	5	ST-41/44 complex/Lineage 3	B				TGC - AAAAAGAAAATTT	
471	44	5	ST-41/44 complex/Lineage 3	B				TGC - AAAAAGAAAATTT	0,346
477	835	5	ST-41/44 complex/Lineage 3	B				TGC - AAAAAGAAAATTT	
488	835	5	ST-41/44 complex/Lineage 3	B				TGC - AAAAAGAAAATTT	
493	186	5	ST-41/44 complex/Lineage 3	C				TGC - AAAAAGAAAATTT	
500	923	5	ST-41/44 complex/Lineage 3	B				TGC - AAAAAGAAAATTT	
503	44	5	ST-41/44 complex/Lineage 3	B				TGC - AAAAAGAAAATTT	0,300
506	180	5	ST-41/44 complex/Lineage 3	B				TGC - AAAAAGAAAATTT	
523	170	5	ST-41/44 complex/Lineage 3	B				TGC - AAAAAGAAAATTT	
526	170	5	ST-41/44 complex/Lineage 3	B				TGC - AAAAAGAAAATTT	
528	41	5	ST-41/44 complex/Lineage 3	B	7-2	15-39	1-5	TGC - AAAAAGAAAATTT	0,401
535	782	5	ST-41/44 complex/Lineage 3	B				TGC - AAAAAGAAAATTT	
536	782	5	ST-41/44 complex/Lineage 3	B				TGC - AAAAAGAAAATTT	
539	782	5	ST-41/44 complex/Lineage 3	B				TGC - AAAAAGAAAATTT	
545	136	5	ST-41/44 complex/Lineage 3	B				TGC - AAAAAGAAAATTT	
547	41	5	ST-41/44 complex/Lineage 3	B				TGC - AAAAAGAAAATTT	0,504
548	437	5	ST-41/44 complex/Lineage 3	B				TGC - AAAAAGAAAATTT	
555	782	5	ST-41/44 complex/Lineage 3	B				TGC - AAAAAGAAAATTT	
557	783	5	ST-41/44 complex/Lineage 3	B				TGC - AAAAAGAAAATTT	
558	783	5	ST-41/44 complex/Lineage 3	B				TGC - AAAAAGAAAATTT	
559	44	5	ST-41/44 complex/Lineage 3	B				TGC - AAAAAGAAAATTT	0,331
560	782	5	ST-41/44 complex/Lineage 3	B				TGC - AAAAAGAAAATTT	
563	835	5	ST-41/44 complex/Lineage 3	B				TGC - AAAAAGAAAATTT	
568	136	5	ST-41/44 complex/Lineage 3	B				TGC - AAAAAGAAAATTT	
569	41	5	ST-41/44 complex/Lineage 3	B				TGC - AAAAAGAAAATTT	0,221
581	477	5	ST-41/44 complex/Lineage 3	B				TGC - AAAAAGAAAATTT	0,439
582	779	5	ST-41/44 complex/Lineage 3	B				TGC - AAAAAGAAAATTT	
583	136	5	ST-41/44 complex/Lineage 3	B				TGC - AAAAAGAAAATTT	
589	136	5	ST-41/44 complex/Lineage 3	B				TGC - AAAAAGAAAATTT	
590	337	5	ST-41/44 complex/Lineage 3	C				TGC - AAAAAGAAAATTT	
599	835	5	ST-41/44 complex/Lineage 3	B				TGC - AAAAAGAAAATTT	
603	877	5	ST-41/44 complex/Lineage 3					TGC - AAAAAGAAAATTT	
608	839	5	ST-41/44 complex/Lineage 3	B				TGC - AAAAAGAAAATTT	
608	839	5	ST-41/44 complex/Lineage 3	B				TGC - AAAAAGAAAATTT	
613	836	5	ST-41/44 complex/Lineage 3	B				TGC - AAAAAGAAAATTT	
614	835	5	ST-41/44 complex/Lineage 3	B				TGC - AAAAAGAAAATTT	

615	44	5	ST-41/44 complex/Lineage 3	B				TGC - AAAAAGAAAATTT	0,360	
620	180	5	ST-41/44 complex/Lineage 3	B				TGC - AAAAAGAAAATTT		
627	43	5	ST-41/44 complex/Lineage 3	cnl				TGC - AAAAAGAAAATTT		
640	337	5	ST-41/44 complex/Lineage 3	C				TGC - AAAAAGAAAATTT		
645	112	5	ST-41/44 complex/Lineage 3	B				TGC - AAAAAGAAAATTT		
646	44	5	ST-41/44 complex/Lineage 3	B				TGC - AAAAAGAAAATTT		
649	833	5	ST-41/44 complex/Lineage 3	B				TGC - AAAAAGAAAATTT		
650	41	5	ST-41/44 complex/Lineage 3	B				TGC - AAAAAGAAAATTT	0,293	
653	973	5	ST-41/44 complex/Lineage 3	B				TGC - AAAAAGAAAATTT		
656	136	5	ST-41/44 complex/Lineage 3	B				TGC - AAAAAGAAAATTT		
665	839	5	ST-41/44 complex/Lineage 3	B				TGC - AAAAAGAAAATTT		
671	787	5	ST-41/44 complex/Lineage 3	B				TGC - AAAAAGAAAATTT		
675	908	5	ST-41/44 complex/Lineage 3	B				TGC - AAAAAGAAAATTT		
681	437	5	ST-41/44 complex/Lineage 3	B				TGC - AAAAAGAAAATTT		
696	44	5	ST-41/44 complex/Lineage 3	B				TGC - AAAAAGAAAATTT		
708	44	5	ST-41/44 complex/Lineage 3	B				TGC - AAAAAGAAAATTT		
725	44	5	ST-41/44 complex/Lineage 3	B				TGC - AAAAAGAAAATTT		
726	41	5	ST-41/44 complex/Lineage 3	B				TGC - AAAAAGAAAATTT	0,339	
790	280	5	ST-41/44 complex/Lineage 3	B				AGC - AAAAAGAAAATTT		
808	303	5	ST-41/44 complex/Lineage 3	B				TGC - AAAAAGAAAATTT		
808	303	5	ST-41/44 complex/Lineage 3	B				TGC - AAAAAGAAAATTT		
828	44	5	ST-41/44 complex/Lineage 3	B				TGC - AAAAAGAAAATTT		
343	41	6	ST-41/44 complex/Lineage 3	B				TGCAAAAAAGAAAATTT	1,011	
356	41	6	ST-41/44 complex/Lineage 3	B				TGCAAAAAAGAAAATTT	0,624	
454	1127	6	ST-41/44 complex/Lineage 3	B				TGCAAAAAAGAAAATTT	0,772	
480	778	6	ST-41/44 complex/Lineage 3	B				TGCAAAAAAGAAAATTT	-0,011	deletion at position of bp193

overview disease strains (DE-strains from German National Reference Centre for Meningococci (NRZM))

strain (DE)	sequence type (ST)	No of 'A' in poly-A stretch	clonal complex	serogroup (genotype)	PorA (VR1)	PorA (VR2)	fetA	sequence poly-A stretch in *nspA* promoter	NspA-ELISA (OD)	
8397	2287	5	ST-41/44 complex/Lineage 3	B	19-1	13-1	1-7	TGC - AAAAAGAAAATTT		
8403	6944	6	ST-41/44 complex/Lineage 3	B	5-2	10-1	5-7	TGCAAAAAAGAAAATTT		
8519	2748	5	ST-41/44 complex/Lineage 3	B	5-1	10-1	1-5	TGC - AAAAAGAAAATTT		
8521	2713	5	ST-41/44 complex/Lineage 3	B	12-1	13-1	1-5	TGC - AAAAAGAAAATTT		
8577	6945	5	ST-41/44 complex/Lineage 3	B	7-2	4	1-5	TGC - AAAAAGAAAATTT	0,313	
8578	1374	6	ST-41/44 complex/Lineage 3	B	7-2	13-9	4-1	TGCAAAAAAGAAAATTT		
8623	303	6	ST-41/44 complex/Lineage 3	B	7-2	4	1-5	TGCAAAAAAGAAAATTT		
8627	2827	5	ST-41/44 complex/Lineage 3	B	7-2	4	1-5	TGC - AAAAAGAAAATTT		
8636	6946	6	ST-41/44 complex/Lineage 3	B	7-2	13-2	1-5	TGCAAAAAAGAAAATTT	-0,012	deletion at position of bp193
8646	839	6	ST-41/44 complex/Lineage 3	B	21	16	1-15	TGCAAAAAAGAAAATTTA		
8658	42	6	ST-41/44 complex/Lineage 3	B	7-2	4	1-5	TGCAAAAAAGAAAATTT	1,199	
8674	41	6	ST-41/44 complex/Lineage 3	B	7-2	4	5-12	TGCAAAAAAGAAAATTT	0,855	
8697	839	5	ST-41/44 complex/Lineage 3	B	21	16	1-15	AAC - AAAAAGAAAATTT		
8715	5295	6	ST-41/44 complex/Lineage 3	B	7-2	4	1-5	TGCAAAAAAGAAAATTT		
8740	2491	5	ST-41/44 complex/Lineage 3	B	7-4	4-1	5-2	TGC - AAAAAGAAAATTT		
8761	2016	6	ST-41/44 complex/Lineage 3	B	7-2	13-2	1-5	TGCAAAAAAGAAAATTT		
8792	1145	5	ST-41/44 complex/Lineage 3	B	7-2	4	1-5	TGC - AAAAAGAAAATTT	0,351	
8794	877	5	ST-41/44 complex/Lineage 3	B	18-7	9-7	4-1	TGC - AAAAAGAAAATTT	0,403	
8832	41	5	ST-41/44 complex/Lineage 3	B	18-1	3	1-5	TGC - AAAAAGAAAATTT	0,224	
8837	43	5	ST-41/44 complex/Lineage 3	B	19	15-1	1-5	TGC - AAAAAGAAAATTT		
8902	303	6	ST-41/44 complex/Lineage 3	B	7-2	4	1-5	TGCAAAAAAGAAAATTT		
8957	2988	5	ST-41/44 complex/Lineage 3	B	7-2	4	3-3	TGC - AAAAAGAAAATTT		
8958	3477	6	ST-41/44 complex/Lineage 3	B	7-2	4	1-5	TGCAAAAAAGAAAATTT		
8963	40	5	ST-41/44 complex/Lineage 3	B	5-2	10-1	5-7	TGCAAAAAAGAAAATTT	0,667	
8973	207	5	ST-41/44 complex/Lineage 3	B	7-4	1	1-5	TGC - AAAAAGAAAATTT		
8974	2821	5	ST-41/44 complex/Lineage 3	B	19	15-1	1-5	TGC - AAAAAGAAAATTT		
9021	3021	6	ST-41/44 complex/Lineage 3	B	7-2	4	4-3	TGCAAAAAAGAAAATTT		
9044	2799	5	ST-41/44 complex/Lineage 3	B	22	26	4-1	TGC - AAAAAGAAAATTT		
9072	159	6	ST-41/44 complex/Lineage 3	B	7-2	4	1-5	TGCAAAAAAGAAAATTT		
9077	5115	6	ST-41/44 complex/Lineage 3	B	7-2	4	3-9	TGCAAAAAAGAAAATTT		
9113	3022	6	ST-41/44 complex/Lineage 3	B	7-2	4	1-5	TGCAAAAAAGAAAATTT	0,818	
9128	2916	5	ST-41/44 complex/Lineage 3	B	7-2	4	1-5	TGC - AAAAAGAAAATTT		
9129	41	6	ST-41/44 complex/Lineage 3	B	7-2	4	1-5	TGCAAAAAAGAAAATTT	0,888	
9133	280	5	ST-41/44 complex/Lineage 3	B	18-1	3	1-5	TGC - AAAAAGAAAATTT		
9141	3478	5	ST-41/44 complex/Lineage 3	B	18	25	5-43	TGC - AAAAAGAAAATTT	0,230	
9185	1475	5	ST-41/44 complex/Lineage 3	B	7-2	4	3-5	TGC - AAAAAGAAAATTT		
9198	2253	6	ST-41/44 complex/Lineage 3	B	7-2	4	1-5	TGCAAAAAAGAAAATTT		
9267	1475	5	ST-41/44 complex/Lineage 3	B	7-2	4	4-1	TGC - AAAAAGAAAATTT		
9290	6739	5	ST-41/44 complex/Lineage 3	B	7-4	1	1-5	TGC - AAAAAGAAAATTT		
9321	42	5	ST-41/44 complex/Lineage 3	B	7-2	4	1-28	TGC - AAAAAGAAAATTT	0,278	
9331	477	6	ST-41/44 complex/Lineage 3	B	18	25	1-5	TGCAAAAAAGAAAATTT	0,997	
9359	280	5	ST-41/44 complex/Lineage 3	B	18-1	3	1-5	TGC - AAAAAGAAAATTT		
9379	482	6	ST-41/44 complex/Lineage 3	B	7-2	4	1-5	TGCAAAAAAGAAAATTT	0,848	
9469	6948	5	ST-41/44 complex/Lineage 3	B	19	15-1	1-5	TGC - AAAAAGAAAATTT		
9484	5444	5	ST-41/44 complex/Lineage 3	B	22	14	1-5	TGC - AAAAAGAAAATTT		
9492	41	5	ST-41/44 complex/Lineage 3	B	7-2	4	1-5	TGC - AAAAAGAAAATTT	0,456	
9499	318	6	ST-41/44 complex/Lineage 3	B	7-2	4	1-5	TGCAAAAAAGAAAATTT		
9509	6949	6	ST-41/44 complex/Lineage 3	B	7-2	4	4-7	TGCAAAAAAGAAAATTT		
9514	2764	6	ST-41/44 complex/Lineage 3	B	22	9	1-5	TGCAAAAAAGAAAATTT	0,854	
9547	6740	5	ST-41/44 complex/Lineage 3	B	7-1	1	5-28	TGC - AAAAAGAAAATTT		
9563	6741	5	ST-41/44 complex/Lineage 3	B	22	14-6	1-5	TGC - AAAAAGAAAATTT		
9584	191	5	ST-41/44 complex/Lineage 3	B	21	16	1-5	TGC - AAAAAGAAAATTT		
9593	6949	6	ST-41/44 complex/Lineage 3	B	18-1	3	1-5	TGCAAAAAAGAAAATTT		
9666	6742	5	ST-41/44 complex/Lineage 3	B	7-2	16	1-5	TGC - AAAAAGAAAATTT		
9678	154	5	ST-41/44 complex/Lineage 3	B	7-2	4	1-5	TGC - AAAAAGAAAATTT		
9686	42	6	ST-41/44 complex/Lineage 3	B	7-2	4	1-5	TGCAAAAAAGAAAATTT	0,912	
9689	2713	5	ST-41/44 complex/Lineage 3	B	12-1	13-2	1-5	TGC - AAAAAGAAAATTT		
9691	6950	6	ST-41/44 complex/Lineage 3	B	7-2	2-24	5-7	TGCAAAAAAGAAAATTT		
9699	6944	6	ST-41/44 complex/Lineage 3	B	18-1	3	1-5	TGCAAAAAAGAAAATTT	-0,058	deletion at position of bp193
9708	6944	6	ST-41/44 complex/Lineage 3	B	7-2	13-2	1-5	TGCAAAAAAGAAAATTT		
9711	6944	6	ST-41/44 complex/Lineage 3	B	5-1	10-8	1-5	TGCAAAAAAGAAAATTT		
9730	112	5	ST-41/44 complex/Lineage 3	B	5-1	2-2	1-14	TGC - AAAAAGAAAATTT		
9745	2764	5	ST-41/44 complex/Lineage 3	B	22	9	1-5	TGC - AAAAAGAAAATTT	0,149	
9751	2082	5	ST-41/44 complex/Lineage 3	B	21	16	5-8	TGC - AAAAAGAAAATTT		

9784	42	6	ST-41/44 complex/Lineage 3	B	7-2	4	1-5	TGCAAAAAAGAAAATTT	0,871	
9834	6947	6	ST-41/44 complex/Lineage 3	B	5-1	10-8	1-5	TGCAAAAAAGAAAATTT		
9874	1194	5	ST-41/44 complex/Lineage 3	B	19	15	1-5	TGC-AAAAAGAAAATTT	0,349	
9890	6944	6	ST-41/44 complex/Lineage 3	B	7-2	2-24	5-7	TGCAAAAAAGAAAATTT		
10009	280	5	ST-41/44 complex/Lineage 3	B	18-1	3	1-5	TGC-AAAAAGAAAATTT		
10049	41	6	ST-41/44 complex/Lineage 3	B	7-2	4	1-5	TGCAAAAAAGAAAATTT	0,812	
10074	42	6	ST-41/44 complex/Lineage 3	B	7-2	4	1-5	TGCAAAAAAGAAAATTT	0,771	
10084	4489	5	ST-41/44 complex/Lineage 3	B	22	14-6	1-5	TGC-AAAAAGAAAATTT		
10096	1127	6	ST-41/44 complex/Lineage 3	B	5-1	10-8	1-5	TGCAAAAAAGAAAATTT	-0,023	deletion at position of bp193
10111	4796	6	ST-41/44 complex/Lineage 3	B	7-2	4	1-5	TGCAAAAAAGAAAATTT		
10120	4972	6	ST-41/44 complex/Lineage 3	B	7-2	4	1-5	TGCAAAAAAGAAAATTT		
10201	6952	5	ST-41/44 complex/Lineage 3	B	17	16-4	1-5	TGC-AAAAAGAAAATTT		
10222	112	5	ST-41/44 complex/Lineage 3	B	5-1	2-2	1-14	TGC-AAAAAGAAAATTT		
10241	5572	5	ST-41/44 complex/Lineage 3	B	22	14-6	1-5	TGC-AAAAAGAAAATTT		
10315	280	5	ST-41/44 complex/Lineage 3	B	18-1	3	1-5	AGC-AAAAAGAAAATTT		
10465	136	5	ST-41/44 complex/Lineage 3	B	17	16-3	5-5	TGC-AAAAAGAAAATTT		
10470	42	6	ST-41/44 complex/Lineage 3	B	7-2	4	1-5	TGCAAAAAAGAAAATTT	1,003	
10479	2713	5	ST-41/44 complex/Lineage 3	B	12-1	13-9	1-5	TGC-AAAAAGAAAATTT		
10488	41	6	ST-41/44 complex/Lineage 3	B	7-2	4	1-5	TGCAAAAAAGAAAATTT	0,977	
10520	43	6	ST-41/44 complex/Lineage 3	B	12-1	1	5-47	TGCAAAAAAGAAAATTT		
10625	6953	5	ST-41/44 complex/Lineage 3	B	18-1	3	1-7	TGC-AAAAAGAAAATTT		
10648	5737	6	ST-41/44 complex/Lineage 3	B	7-2	4	1-5	TGCAAAAAAGAAAATTT		
10690	4736	6	ST-41/44 complex/Lineage 3	B	7-2	4	1-5	TGCAAAAAAGAAAATTT		
10775	6954	5	ST-41/44 complex/Lineage 3	B	5-2	10	1-7	TGC-AAAAAGAAAATTT		
10789	3521	5	ST-41/44 complex/Lineage 3	B	7-2	30	1-5	TGC-AAAAAGAAAATTT		
10797	1475	5	ST-41/44 complex/Lineage 3	B	7-2	4	5-8	TGC-AAAAAGAAAATTT		
10829	477	5	ST-41/44 complex/Lineage 3	B	7-2	4	1-5	TGC-AAAAAGAAAATTT	0,287	
10844	42	6	ST-41/44 complex/Lineage 3	B	7-2	4	1-5	TGCAAAAAAGAAAATTT	0,963	
10882	112	5	ST-41/44 complex/Lineage 3	B	5-1	2-2	1-14	TGC-AAAAAGAAAATTT		
10961	280	6	ST-41/44 complex/Lineage 3	B	18-1	3	1-5	AGC-AAAAAGAAAATTT	0,278	
10985	571	5	ST-41/44 complex/Lineage 3	B	18-7	9	1-7	TGC-AAAAAGAAAATTT		
11120	3346	5	ST-41/44 complex/Lineage 3	C	21-2	28	5-5	TGC-AAAAAGAAAATTT		
11126	42	6	ST-41/44 complex/Lineage 3	B	7-2	4	1-5	TGCAAAAAAGAAAATTT	0,920	
11138	6441	5	ST-41/44 complex/Lineage 3	B	12-1	13-1	5-5	TGC-AAAAAGAAAATTT		
11147	3050	5	ST-41/44 complex/Lineage 3	B	7-2	4	1-5	TGC-AAAAAGAAAATTT		
11150	485	5	ST-41/44 complex/Lineage 3	B	7-1	1	1-14	TGC-AAAAAGAAAATTT		
11151	8333	5	ST-41/44 complex/Lineage 3	B	7-2	13-2	1-5	TGC-AAAAAGAAAATTT	0,215	
11154	3346	5	ST-41/44 complex/Lineage 3	C	17	16-4	3-9	TGC-AAAAAGAAAATTT		
11169	1834	5	ST-41/44 complex/Lineage 3	B	7-1	1	1-5	TGC-AAAAAGAAAATTT		
11170	8336	5	ST-41/44 complex/Lineage 3	B	18	10-1	1-5	TGC-AAAAAGAAAATTT		
11175	40	5	ST-41/44 complex/Lineage 3	B	7-2	13-2	1-5	TGC-AAAAAGAAAATTT	0,216	
11183	8340	6	ST-41/44 complex/Lineage 3	B	7-2	13-9	1-5	TGCAAAAAAGAAAATTT		
11188	8344	6	ST-41/44 complex/Lineage 3	B	7-2	4	1-5	TGCAAAAAAGAAAATTT		
11192	2764	6	ST-41/44 complex/Lineage 3	B	7-2	4	1-5	TGCAAAAAAGAAAATTT	0,778	
11198	8348	6	ST-41/44 complex/Lineage 3	B	7-2	13-9	1-5	TGCAAAAAAGAAAATTT		
11202	8350	5	ST-41/44 complex/Lineage 3	B	18-1	3	1-5	TGC-AAAAAGAAAATTT	0,428	
11204	5460	6	ST-41/44 complex/Lineage 3	B	7-2	4	1-5	TGCAAAAAAGAAAATTT	0,925	
11205	8351	5	ST-41/44 complex/Lineage 3	B	18-7	26	5-72	TGC-AAAAAGAAAATTT		
11216	8355	5	ST-41/44 complex/Lineage 3	B	18-1	3	1-5	TGC-AAAAAGAAAATTT	0,414	
11217	691	5	ST-41/44 complex/Lineage 3	B	5-1	10-6	1-7	TGC-AAAAAGAAAATTT		
11220	8356	5	ST-41/44 complex/Lineage 3	B	21	16	1-5	TGC-AAAAAGAAAATTT		
11229	2203	6	ST-41/44 complex/Lineage 3	B	7-2	4	1-5	TGCAAAAAAGAAAATTT		
11232	691	5	ST-41/44 complex/Lineage 3	B	7-1	1	1-7	TGC-AAAAAGAAAATTT		
11240	8362	5	ST-41/44 complex/Lineage 3	B	7-1	1	1-7	TGC-AAAAAGAAAATTT		
11272	41	5	ST-41/44 complex/Lineage 3	B	18-1	3	5-8	TGC-AAAAAGAAAATTT	0,291	
11285	839	5	ST-41/44 complex/Lineage 3	B	21	16	1-15	AAC-AAAAAGAAAATTA		
11287	839	5	ST-41/44 complex/Lineage 3	B	21	16	1-15	AAC-AAAAAGAAAATTT	0,232	
11300	112	5	ST-41/44 complex/Lineage 3	B	21-3	9	1-14	TGC-AAAAAGAAAATTT		
11321	318	5	ST-41/44 complex/Lineage 3	B	5-2	10-11	5-2	TGC-AAAAAGAAAATTT		
11345	973	5	ST-41/44 complex/Lineage 3	B	19-1	13	5-5	TGC-AAAAAGAAAATTT		
11367	112	5	ST-41/44 complex/Lineage 3	B	5-1	2-2	1-14	TGC-AAAAAGAAAATTT		
11381	41	6	ST-41/44 complex/Lineage 3	B	7-2	4	1-5	TGCAAAAAAGAAAATTT	0,878	
11390	191	5	ST-41/44 complex/Lineage 3	B	22	14-6	3-6	TGC-AAAAAGAAAATTT		
11412	112	5	ST-41/44 complex/Lineage 3	B	5-1	2-2	1-14	TGC-AAAAAGAAAATTT		
11467	42	6	ST-41/44 complex/Lineage 3	B	7-2	10-2	1-5	TGCAAAAAAGAAAATTT	0,780	
11470	944	5	ST-41/44 complex/Lineage 3	B	19	15	3-9	TGC-AAAAAGAAAATTT		
11553	839	5	ST-41/44 complex/Lineage 3	B	19	15-1	1-15	AGC-AAAAAGAAAATTT		
11576	437	5	ST-41/44 complex/Lineage 3	C	5-1	2-59	5-2	TGC-AAAAAGAAAATTT		
11584	180	6	ST-41/44 complex/Lineage 3	B	7-2	13-2	1-5	TGCAAAAAAGAAAATTT		
11606	41	5	ST-41/44 complex/Lineage 3	B	7-2	4	1-5	TGC-AAAAAGAAAATTT	0,832	
11635	318	5	ST-41/44 complex/Lineage 3	B	22	14	1-5	TGC-AAAAAGAAAATTT		
11704	41	6	ST-41/44 complex/Lineage 3	B	19	15	1-5	TGCAAAAAAGAAAATTT	0,765	
12143	839	5	ST-41/44 complex/Lineage 3	B	19	15-1	1-15	TGC-AAAAAGAAAATTT		

supplementary table S3: ***N. meningitidis*** **strains with '6A' in NspA Promotor (from BLAST-serach of PubMLST)**

PubMLST-	isolate	ST	cc	serogroup
409	BZ 198	ST-41	cc41/44	B
646	400	ST-40	cc41/44	B
651	931905	ST-41	cc41/44	B
653	91/40	ST-42	cc41/44	B
2914	OX0051332	ST-1957	cc41/44	cnl
19263	NZ-05/33	ST-42	cc41/44	B
19960	M10 240500	ST-41	cc41/44	B
20005	M10 240589	ST-40	cc41/44	B
20008	M10 240592	ST-6782	cc41/44	B
20011	M10 240598	ST-41	cc41/44	B
20034	M10 240634	ST-42	cc41/44	B
20063	M10 240681	ST-9883	cc41/44	B
20092	M10 240722	ST-41	cc41/44	B
20109	M10 240750	ST-8203	cc41/44	B
20134	M10 240785	ST-3447	cc41/44	B
20140	M10 240793	ST-6782	cc41/44	B
20142	M10 240796	ST-136	cc41/44	B
20174	M11 240011	ST-42	cc41/44	B
20212	M11 240053	ST-41	cc41/44	B
20253	M11 240112	ST-8052	cc41/44	B
20327	M11 240244	ST-1960	cc41/44	B
20788	M10 240115	ST-41	cc41/44	B
20886	M01 240245	ST-42	cc41/44	B
20887	M01 240258	ST-42	cc41/44	B
20915	M01 241920	ST-42	cc41/44	B
20916	M01 241930	ST-42	cc41/44	B
21095	M11 240447	ST-1960	cc41/44	B
21149	M11 240600	ST-9246	cc41/44	B
21184	M11 240766	ST-8054	cc41/44	B
21188	M11 240773	ST-41	cc41/44	B
21234	M11 241013	ST-41	cc41/44	B
21291	M12 240009	ST-41	cc41/44	B
21321	M12 240045	ST-41	cc41/44	B
21337	M12 240071	ST-10278	cc41/44	B
21340	M12 240077	ST-41	cc41/44	B
21355	M12 240097	ST-41	cc41/44	B
21411	M12 240186	ST-6782	cc41/44	B
21455	M12 240252	ST-2203	cc41/44	B
21496	M12 240321	ST-1403	cc41/44	B
26293	BB238	ST-41	cc41/44	cnl
26378	BB291	ST-41	cc41/44	cnl
26463	R291	ST-41	cc41/44	cnl
26485	R304	ST-41	cc41/44	cnl
26492	T291	ST-41	cc41/44	cnl
26836	12003_2011	ST-41	cc41/44	B
26865	12002_2012	ST-1403	cc41/44	B
26875	12012_2012	ST-206	cc41/44	B
26890	12031_2012	ST-10132	cc41/44	B
26893	12036_2012	ST-1403	cc41/44	B
27792	M12 240658	ST-42	cc41/44	B
27909	M13 240009	ST-2799	cc41/44	B
27929	M13 240047	ST-8054	cc41/44	B
27944	M13 240085	ST-40	cc41/44	B
27983	M13 240173	ST-4256	cc41/44	B
27984	M13 240174	ST-2203	cc41/44	B
28869	OX9931039	ST-41	cc41/44	B
28871	OX9931066	ST-41	cc41/44	B
28885	OX9931715	ST-41	cc41/44	B
28893	OX9932020	ST-41	cc41/44	B
28901	OX0050209	ST-41	cc41/44	cnl
28903	OX0050304	ST-41	cc41/44	B
28937	OX01060954	ST-3259	cc41/44	B
28956	OX01062098	ST-1957	cc41/44	cnl
28966	OX9930855	ST-180	cc41/44	B
29767	M00 240858	ST-41	cc41/44	B
29768	M00 240859	ST-41	cc41/44	B
29769	M00 240860	ST-41	cc41/44	B
29770	M00 240863	ST-42	cc41/44	B
29771	M00 240877	ST-42	cc41/44	B
29772	M00 240878	ST-42	cc41/44	B
30115	M99 242419	ST-41	cc41/44	B
30116	M99 242434	ST-41	cc41/44	B
30117	M99 242435	ST-41	cc41/44	B
31176	NM8250	ST-8052	cc41/44	B
31190	NM9062	ST-41	cc41/44	B
31196	NM9853	ST-41	cc41/44	B
31196	NM9853	ST-41	cc41/44	B
31207	NM10833	ST-10867	cc41/44	B
34542	NZ98/254	ST-42	cc41/44	B
35255	09.2522.Q	ST-42	cc41/44	B
35299	11.1312.X	ST-42	cc41/44	B
35390	13.6161.J	ST-42	cc41/44	B
35550	M14 240018	ST-6782	cc41/44	B
35556	M14 240027	ST-1403	cc41/44	B

(continued)

PubMLST-	isolate	ST	cc	serogroup
40442	LNP27525	ST-41	cc41/44	B
40487	LNP28294	ST-41	cc41/44	B
41322	LIM7761	ST-4960	cc41/44	B
41655	2751	ST-1403	cc41/44	B
41692	84Mo	ST-1403	cc41/44	B
41730	LNP28438	ST-1403	cc41/44	B
41805	LNP28452	ST-41	cc41/44	B
41968	16-102	ST-44	cc41/44	B
42049	LNP28483	ST-13440	cc41/44	B
42111	IPM1	ST-1255	cc41/44	B
42114	IPM4	ST-12010	cc41/44	B
42115	IPM5	ST-1255	cc41/44	B
42197	LNP28498	ST-1403	cc41/44	B
42219	IPM74	ST-41	cc41/44	B
42453	M16 240080	ST-41	cc41/44	cnl
42650	15-8708141	ST-42	cc41/44	B
42726	CR7104	ST-42	cc41/44	B
42727	CR1994	ST-42	cc41/44	B
42859	1988	ST-1403	cc41/44	discrepancy
42876	LNP28565	ST-8503	cc41/44	B
45332	LNP28669	ST-12347	cc41/44	B
46978	DE8669	ST-42	cc41/44	B
47014	B6116/77	ST-42	cc41/44	B
47261	M16 240512	ST-1403	cc41/44	B
47318	M16 240645	ST-2203	cc41/44	B
51289	B317	ST-41	cc41/44	cnl
51446	R937	ST-41	cc41/44	cnl
51857	W-5959	ST-41	cc41/44	B
51860	W-5981	ST-41	cc41/44	B
53063	M16 240720	ST-41	cc41/44	B
53126	M16 240804	ST-3754	cc41/44	B
53209	M17 240007	ST-41	cc41/44	B
53265	M17 240069	ST-3754	cc41/44	B
57302	BF0689	ST-206	cc41/44	C
57303	BF0692	ST-206	cc41/44	C
57304	BF0751	ST-206	cc41/44	C
57305	BF0791	ST-7929	cc41/44	C
57306	BF1626	ST-206	cc41/44	C
57309	BF2880	ST-206	cc41/44	C
60973	M18 240047	ST-41	cc41/44	B
61009	M17 240805	ST-2203	cc41/44	B
398	BZ 10	ST-8	cc8	B
407	BZ 163	ST-9	cc8	B
640	SB25	ST-8	cc8	C
641	94/155	ST-66	cc8	C
642	312 901	ST-8	cc8	C
20979	M98 250319	ST-153	cc8	B
20980	M98 250320	ST-153	cc8	B
26881	12021_2012	ST-8	cc8	B
26882	12022_2012	ST-8	cc8	B
29645	M98 251974	ST-8	cc8	C
29662	M00 240085	ST-11159	cc8	C
29926	M99 243956	ST-8	cc8	C
30218	78693	ST-9	cc8	W
30219	78786	ST-9	cc8	W
35377	12.8444.A	ST-8	cc8	C
39838	M13970	ST-8	cc8	C
39843	M08566	ST-66	cc8	C
39848	M05721	ST-8	cc8	C
39853	M05726	ST-66	cc8	C
39856	M05729	ST-8	cc8	C
57559	12151-00	ST-8	cc8	C
468	BRAZ 10	ST-11	cc11	C
8682	0030/01	ST-11	cc11	B
29641	M08 240231	ST-7979	cc11	C
29646	M98 253000	ST-11	cc11	C
29901	M98 250863	ST-11	cc11	C
30240	F85 0000045	ST-11	cc11	C
30241	F85 0000076	ST-11	cc11	C
34571	73704	ST-11	cc11	C
34577	87255	ST-10308	cc11	B
34669	NM94	ST-11	cc11	C
37940	M15 240193	ST-11	cc11	W
39837	M05730	ST-11	cc11	C
42720	CR179	ST-11	cc11	C
57534	12014-02	ST-11	cc11	C
57831	0008/17	ST-11	cc11	C
21577	SA_serogroup A_NM5	ST-1	cc1	A
21579	SA_serogroup A_NM7	ST-1	cc1	A
21580	SA_serogroup A_NM8	ST-1	cc1	A
21585	SA_serogroup	ST-1	cc1	A
21586	SA_serogroup	ST-1	cc1	A
40607	11483	ST-1	cc1	C
60554	46	ST-75	cc1	A

35585	M14 240096	ST-3447	cc41/44	B
35735	M14 240357	ST-41	cc41/44	B
35789	M14 240471	ST-8054	cc41/44	B
35790	M14 240472	ST-8054	cc41/44	B
35793	M14 240475	ST-11309	cc41/44	B
36204	BM59	ST-41	cc41/44	B
36205	BM59a	ST-41	cc41/44	B
36206	BM59b	ST-41	cc41/44	B
36207	BM59c	ST-41	cc41/44	B
36225	OX9931966	ST-41	cc41/44	B
37724	M14 240564	ST-41	cc41/44	B
38056	M15 240356	ST-41	cc41/44	B
38094	M15 240474	ST-2203	cc41/44	B
38135	M01 241601	ST-42	cc41/44	B
39383	M15 240759	ST-8054	cc41/44	B
39963	LNP27359	ST-1403	cc41/44	B
39970	LNP27374	ST-1403	cc41/44	B
39976	LNP27388	ST-41	cc41/44	B
39993	LNP27432	ST-41	cc41/44	B
40027	LNP27537	ST-1403	cc41/44	B
40039	LNP27567	ST-41	cc41/44	B
40051	LNP27588	ST-2925	cc41/44	B
40061	LNP27613	ST-207	cc41/44	B
40100	LNP27322	ST13311	cc41/44	B
40166	LNP27392	ST-41	cc41/44	B
40166	LNP27392	ST-41	cc41/44	B
40176	LNP27347	ST-41	cc41/44	B
40177	LNP27348	ST-11826	cc41/44	B
40183	LNP27332	ST-42	cc41/44	B
40185	LNP27466	ST-1403	cc41/44	B
40188	LNP27325	ST-9246	cc41/44	B
40191	LNP27311	ST-11749	cc41/44	B
40240	LNP28305	ST-191	cc41/44	B
40244	LNP28310	ST-5446	cc41/44	B
40338	2582	ST-1403	cc41/44	B
40348	2661	ST-11851	cc41/44	B
40394	LNP28366	ST-4893	cc41/44	B
40398	LNP28370	ST-41	cc41/44	B

60691	37	ST-3349	cc1	A
60841	38	ST-3349	cc1	A
61195	M1755/18	ST-75	cc1	A
61350	Nm-81	ST-75	cc1	A
2216	0190/93	ST-998	cc269	B
27840	M12 240765	ST-2738	cc269	B
41485	M15 240895	ST-8616	cc269	B
45343	LNP28685	ST-6416	cc269	B
56702	NLMB174	ST-13	cc269	B
56704	NLMB192	ST-13	cc269	B
56718	NLMB069	ST-13	cc269	B
57248	NMLB069	ST-13	cc269	B
20347	M11 240278	ST-1655	cc23	Y
26097	159000130	ST-23	cc23	Y
26103	159000341	ST-23	cc23	Y
26170	1059000188	ST-1655	cc23	Y
52876	168.708.601	ST-1946	cc461	B
53378	16-8708600	ST-1946	cc461	B
53379	16-8708601	ST-1946	cc461	B
44647	107530	ST-213	cc213	B
53068	M16 240728	ST-12985	cc213	B
20413	M11 240371	ST-162	cc162	B
21309	M12 240031	ST-162	cc162	B
52813	2586	ST-5770	cc175	C
47096	PT37	ST-823	cc198	cnl
41663	2760	ST-1157	cc1157	discrepancy
1893	0003/93	ST-678	no cc	C
34574	77221	ST-9798	no cc	A
37900	M15 240139	ST-863	no cc	B
38228	107506	ST-10184	no cc	B
38229	107508	ST-10184	no cc	B
40174	LNP27342	ST-11825	no cc	B
60888	M18 240027	ST-18311	no cc	B
28302	N59.5	not typed	not typed	not typed
34670	NM95	not typed	not typed	C
35671	M14 240254	not typed	not typed	B
35764	M14 240407	not typed	not typed	B
42776	LNP28557	not typed	not typed	C

supplementary table S4: *N. meningitidis* mutants used in this study

strain name	WUE number	parental strain	mutation 1	plasmid (#)	resistance	mutation 2	plasmid (#)	resistance	mutation 3	plasmid (#)	resistance	mutation 4	plasmid (#)	resistance
α16Δ*fhbp*Δ*csb*	4234	α16	ΔfHbp	pMP1 (#4170)	Spec	Δcsb	pMF32.35::T5 (#43)	Cm						
DE9686Δ*fhbp*Δ*csb*	4240	DE9686	ΔfHbp	pMP1 (#4170)	Spec	Δcsb	pMF32.35::T5 (#43)	Cm						
α16Δ*fhbp*Δ*csb*Δ*lst*	4294	α16	ΔfHbp	pMP1 (#4170)	Spec	Δcsb	pMF32.35::T5 (#43)	Cm	Δlst	pGH7 (#2326)	Kana			
α528Δ*fhbp*Δ*csb*Δ*lst*	4297	α528	ΔfHbp	pMP1 (#4170)	Spec	Δcsb	pMF32.35::T5 (#43)	Cm	Δlst	pGH7 (#2326)	Kana			
DE9686Δ*fhbp*Δ*csb*Δ*lst*	4300	DE9686	ΔfHbp	pMP1 (#4170)	Spec	Δcsb	pMF32.35::T5 (#43)	Cm	Δlst	pGH7 (#2326)	Kana			
DE9686Δ*fhbp*Δ*csb*Δ*nspA*	4791	DE9686	ΔfHbp	pMP1 (#4170)	Spec	Δcsb	pMF32.35::T5 (#43)	Cm	ΔnspA	pAB13 (#4789)	Ery			
DE9686Δ*fhbp*Δ*csb*Δ*nspA*c*nspA*	4794	DE9686	ΔfHbp	pMP1 (#4170)	Spec	Δcsb	pMF32.35::T5 (#43)	Cm	ΔnspA	pAB13 (#4789)	Ery	nspA+	pAB14 (#4793)	Kana
α16Δ*fhbp*Δ*csb*Δ*nspA*	4947	α16	ΔfHbp	pMP1 (#4170)	Spec	Δcsb	pMF32.35::T5 (#43)	Cm	ΔnspA	pAB13 (#4789)	Ery			
α16Δ*fhbp*Δ*csb* '5A'	4956	α16	ΔfHbp	pMP1 (#4170)	Spec	Δcsb	pMF32.35::T5 (#43)	Cm	nspA ‘5A‘	(PCR product)	Kana			
α16Δ*fhbp*Δ*csb* '6A'	4957	α16	ΔfHbp	pMP1 (#4170)	Spec	Δcsb	pMF32.35::T5 (#43)	Cm	nspA ’6A’	(PCR product)	Kana			
DE9686Δ*fhbp*Δ*csb* '5A'	4958	DE9686	ΔfHbp	pMP1 (#4170)	Spec	Δcsb	pMF32.35::T5 (#43)	Cm	nspA ‘5A‘	(PCR product)	Kana			
DE9686Δ*fhbp*Δ*csb* '6A'	4959	DE9686	ΔfHbp	pMP1 (#4170)	Spec	Δcsb	pMF32.35::T5 (#43)	Cm	nspA ’6A’	(PCR product)	Kana			
DE9686Δ*fhbp*Δ*csb* '5A'Δ*mutS*	4973	DE9686	ΔfHbp	pMP1 (#4170)	Spec	Δcsb	pMF32.35::T5 (#43)	Cm	nspA ‘5A‘	(PCR product)	Kana	Δmut S	pAB7 (#4556)	Ery
α16Δ*nspA*	5466	α16	ΔnspA	pAB13 (#4789)	Ery									
α16Δ*fhbp*	4226	α16	ΔfHbp	pMP1 (#4170)	Spec									
α16Δ*nspA*Δ*fhbp*	5491	α16	ΔfHbp	pMP1 (#4170)	Spec	ΔnspA	pAB13 (#4789)	Ery						
α80Δ*nspA*	5467	α80	ΔnspA	pAB13 (#4789)	Ery									
α80Δ*fhbp*	4227	α80	ΔfHbp	pMP1 (#4170)	Spec									
α80Δ*nspA*Δ*fhbp*	5492	α80	ΔfHbp	pMP1 (#4170)	Spec	ΔnspA	pAB13 (#4789)	Ery						
α253Δ*nspA*	5468	α253	ΔnspA	pAB13 (#4789)	Ery									
α253Δ*fhbp*	4228	α253	ΔfHbp	pMP1 (#4170)	Spec									
α253Δ*nspA*Δ*fhbp*	5493	α253	ΔfHbp	pMP1 (#4170)	Spec	ΔnspA	pAB13 (#4789)	Ery						
α528Δ*nspA*	5469	α528	ΔnspA	pAB13 (#4789)	Ery									
α528Δ*fhbp*	4229	α528	ΔfHbp	pMP1 (#4170)	Spec									
α528Δ*nspA*Δ*fhbp*	5494	α528	ΔfHbp	pMP1 (#4170)	Spec	ΔnspA	pAB13 (#4789)	Ery						
α547Δ*nspA*	5470	α547	ΔnspA	pAB13 (#4789)	Ery									
α547Δ*fhbp*	4230	α547	ΔfHbp	pMP1 (#4170)	Spec									
α547Δ*nspA*Δ*fhbp*	5495	α547	ΔfHbp	pMP1 (#4170)	Spec	ΔnspA	pAB13 (#4789)	Ery						

α726Δ*nspA*	5471	α726	ΔnspA	pAB13 (#4789)	Ery									
α726Δ*fhbp*	4231	α726	ΔfHbp	pMP1 (#4170)	Spec									
α726Δ*nspA*Δ*fhbp*	5496	α726	ΔfHbp	pMP1 (#4170)	Spec	ΔnspA	pAB13 (#4789)	Ery						
DE8794Δ*nspA*	5479	DE8794	ΔnspA	pAB13 (#4789)	Ery									
DE8794Δ*fhbp*	5487	DE8794	ΔfHbp	pMP1 (#4170)	Spec									
DE8794Δ*nspA*Δ*fhbp*	5504	DE8794	ΔfHbp	pMP1 (#4170)	Spec	ΔnspA	pAB13 (#4789)	Ery						
DE9492Δ*nspA*	5480	DE9492	ΔnspA	pAB13 (#4789)	Ery									
DE9492Δ*fhbp*	5488	DE9492	ΔfHbp	pMP1 (#4170)	Spec									
DE9492Δ*nspA*Δ*fhbp*	5505	DE9492	ΔfHbp	pMP1 (#4170)	Spec	ΔnspA	pAB13 (#4789)	Ery						
DE10829Δ*nspA*	5481	DE10829	ΔnspA	pAB13 (#4789)	Ery									
DE10829Δ*fhbp*	5489	DE10829	ΔfHbp	pMP1 (#4170)	Spec									
DE10829Δ*nspA*Δ*fhbp*	5506	DE10829	ΔfHbp	pMP1 (#4170)	Spec	ΔnspA	pAB13 (#4789)	Ery						
DE11216Δ*nspA*	5482	DE11216	ΔnspA	pAB13 (#4789)	Ery									
DE11216Δ*fhbp*	5490	DE11216	ΔfHbp	pMP1 (#4170)	Spec									
DE11216Δ*nspA*Δ*fhbp*	5507	DE11216	ΔfHbp	pMP1 (#4170)	Spec	ΔnspA	pAB13 (#4789)	Ery						
DE8658Δ*nspA*	5475	DE8658	ΔnspA	pAB13 (#4789)	Ery									
DE8658Δ*fhbp*	5484	DE8658	ΔfHbp	pMP1 (#4170)	Spec									
DE8658Δ*nspA*Δ*fhbp*	5500	DE8658	ΔfHbp	pMP1 (#4170)	Spec	ΔnspA	pAB13 (#4789)	Ery						
DE9686Δ*nspA*	5473	DE9686	ΔnspA	pAB13 (#4789)	Ery									
DE9686Δ*fhbp*	4232	DE9686	ΔfHbp	pMP1 (#4170)	Spec									
DE9686Δ*nspA*Δ*fhbp*	5497	DE9686	ΔfHbp	pMP1 (#4170)	Spec	ΔnspA	pAB13 (#4789)	Ery						
DE9129Δ*nspA*	5476	DE9129	ΔnspA	pAB13 (#4789)	Ery									
DE9129Δ*nspA*Δ*fhbp*	5501	DE9129	ΔfHbp	pMP1 (#4170)	Spec	ΔnspA	pAB13 (#4789)	Ery						
DE10470Δ*nspA*	5477	DE10470	ΔnspA	pAB13 (#4789)	Ery									
DE10470Δ*fhbp*	5485	DE10470	ΔfHbp	pMP1 (#4170)	Spec									
DE10470Δ*nspA*Δ*fhbp*	5502	DE10470	ΔfHbp	pMP1 (#4170)	Spec	ΔnspA	pAB13 (#4789)	Ery						
DE11204Δ*nspA*	5478	DE11204	ΔnspA	pAB13 (#4789)	Ery									
DE11204Δ*fhbp*	5486	DE11204	ΔfHbp	pMP1 (#4170)	Spec									
DE11204Δ*nspA*Δ*fhbp*	5503	DE11204	ΔfHbp	pMP1 (#4170)	Spec	ΔnspA	pAB13 (#4789)	Ery						

Table S5: Primers used in this study

primer name	sequence (5'-3')	target gene	amplicon
**construction of mutant strains**			
MP1	GCTCTAGACCAGCCACGCGCATAC	fhbp (upstream)	525 bp
MP6	GCGCGCCAATTGGACGGCATTTTGTTTACAGG		
MP4	CCCGCTCGAGCAGCGTATCGAACCATGC	fhbp (downstream)	567 bp
MP7	GCGCGCCAATTGCGCCAAGCAATAACCATTG		
KH145	GCGCGCTCTAGATTTGAGTTCATGATGAACGCC	nspA (upstream)	790 bp
KH146	GCGCGCGAATTCTTTTGGTTCCTTTATGGTCAGTTAG		
KH147	GCGCGCGAATTCTATGCGCCTTATTCTGCAAAC	nspA (downstream)	520 bp
KH148	GCGCGCCTCGAGTTATGCGTTCAAAACGAGGAC		
**construction of isogenic *nspA* mutants ('5A' versus '6A')**			
HC630	GATACCGCTACGTATCTTGAAGTATTG	127 bp upstream nspA startcodon to 6 bp downstream of nspA stop codon	
HC631	CGCATATCAGAATTTGACGCCATATG		
HC632	AGATCTCCTTATTCTGCAAACCGCC	7bp downstream of nspA stop codon to stop codon of nmb0662	
HC621	TTATGCGTTCAAAACGAGGAC		
**sequencing *nspA* promoter**			
nspA1	GCAACAATCCGACAGATACC		
nspA2	GGTTTGCAGAATAAGGCGCA		
nspA5	GCTTGAGGCTTTTGCGTGTG		
**qRT PCR primer**			
KH124	CCCATCCACCGATTTCAAAC	nspA	69 bp
KH125	GGCGATTGGGTGTCGAAGT		
MP126	GGTATTCGACAGCAGCAAAGC	nmb1567 (endogenous control)	72 bp
MP127	GTCCAACCCGGAATCACTTG		

Restriction sites are underlined
